# Structure elucidation and antioxidant activity of the radical-induced oxidation products obtained from procyanidins B1 to B4

**DOI:** 10.1016/j.crfs.2025.101160

**Published:** 2025-08-09

**Authors:** Annik Fischer, Helen Keller, Jörn Droste, Recep Gök, Tuba Esatbeyoglu

**Affiliations:** a Department of Molecular Food Chemistry and Food Development, Institute of Food and One Health, Leibniz University Hannover, Am Kleinen Felde 30, 30167, Hannover, Germany; b Institute of Organic Chemistry, Leibniz University Hannover, Schneiderberg 1B, 30167, Hannover, Germany; c Institute of Food Chemistry, Technische Universität Braunschweig, Schleinitzstraße 20, 38106, Braunschweig, Germany

**Keywords:** Proanthocyanidin, Radical scavenging activity, Isolation, Countercurrent chromatography, Structure elucidation, Low-temperature NMR, Ion mobility spectrometry

## Abstract

A- and B-type procyanidins (PCs) are widely known for their health-promoting properties, such as antioxidant activity. The limited availability of reference substances represents a major challenge, resulting in a low number of systematic studies on their health benefits. In our study, the optimised 2,2-diphenyl-1-picrylhydrazyl (DPPH) radical-induced oxidation of the B-types B1 and B2 was carried out yielding the corresponding A-types A1 (1) and A2 (2), which have an additional ether bridge between C2–*O*–C7, whereas oxidation of B3 and B4 produced a six-membered spirocyclic ring system including a spiro-carbon atom at C8t (3–5). The compounds (1–5) were fully characterised by combining circular dichroism (CD) and low-temperature NMR spectroscopy at 253.0 K in acetone-*d*_*6*_ including one-dimensional (1D; ^1^H and ^13^C NMR) and two-dimensional (2D; ^1^H–^1^H COSY, ^1^H–^1^H TOCSY, ^1^H–^13^C HSQC, ^1^H–^13^C HMBC, ^1^H–^1^H ROESY) NMR experiments. The collision cross section (CCS) values from the trapped ion mobility spectrometry (TIMS) and time of flight mass spectrometry analysis (TOF-MS) allowed a rapid differentiation of the dimeric A-types, C4→C8-linked B-types, and spiro-linked PCs in combination with their retention times. A comparative analysis of the antioxidant properties of the A-, B-types, and spiro-linked PCs was conducted for the first time. The spiro-linked PCs (3–5) were found to have an equal or even higher antioxidant activity compared to the A-types A1 (1) and A2 (2) and the comparative analysis of the different procyanidin types revealed new insights into their antioxidant properties.

## Highlights

• DPPH radical-induced oxidation of procyanidins (PC) B1–B4 on preparative scale.
• Low-temperature NMR data for A-type and spiro-linked PCs.
• Circular dichroism prediction and measurement for stereochemical verification.
• Rapid differentiation of A-type and spiro-linked PCs by ion mobility.
• Comparison of the antioxidant activity of A-, B-type, and spiro-linked PCs.

## Graphical abstract

**Figure:**
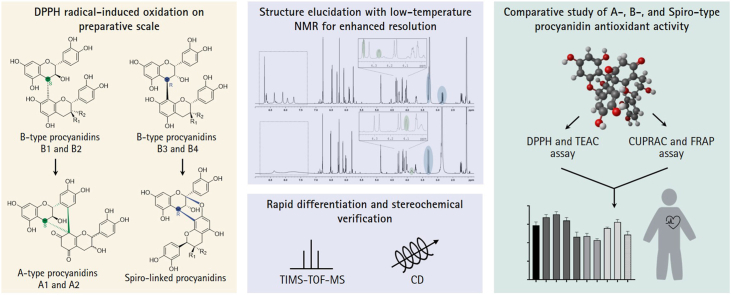

## 1 Introduction

Proanthocyanidins (PAs) are widely found in foods (Gu et al., 2004; Hellström et al., 2009). They are condensation products of flavan-3-ols. Oligomeric PAs include dimers, trimers, and compounds with up to six units, while polymeric PAs contain more than six units (Oliveira et al., 2013). PAs composed of (+)-catechin and (−)-epicatechin units are called procyanidins (PCs). B-type PCs are linked by a C–C interflavan bond. The PC dimers B1–B4, which contain a C4→C8 linkage, are the most abundant PAs in fruit-bearing plants (Haslam, 1982). The C4→C6 linkage of the B5–B8 dimers is less common and naturally occurs in lower amounts (Haslam, 1982; De Bruyne et al., 1999). In addition to the B-type PCs connected by an interflavan bond, there are also A-type PCs linked by an additional C–*O*–C ether bridge between C2 of the upper unit (u) and the oxygen at C7 or C5 of the terminal unit (t) (Oliveira et al., 2013; Zeng et al., 2024).

A-type PCs have been isolated from several plant species, including peanut (*Arachis hypogaea*), lychee (*Litchi chinensis*), and cranberry (*Vaccinium macrocarpon*) (Howell et al., 2005; Appeldoorn et al., 2009; Sui et al., 2021). The most common A-type PCs are A1 and A2. It is possible to isolate these compounds from peanut shells, for example, by employing a two-step high-performance liquid chromatography (HPLC) purification process. Initially, normal phase chromatography is performed to separate the compounds according to their mean degree of polymerisation (mDP). Subsequently, *reversed phase* is used to isolate the A-types (Appeldoorn et al., 2009). They also can be obtained by oxidative conversion of B1 (EC-(4*β*→8)-C) and B2 (EC-(4*β*→8)-EC) in an effective manner (Kondo et al., 2000; Osman and Wong, 2007; Fischer et al., 2025). The conversion from B- to A-type can occur through radical-induced oxidation initiated by 2,2-diphenyl-1-picrylhydrazyl (DPPH) or superoxide anion radical (Kondo et al., 2000; Chen et al., 2014; Jing et al., 2024). Alternatively, enzymatically-induced oxidation involving polyphenol oxidase, laccase, or xanthine oxidase can be used (Osman and Wong, 2007; Chen et al., 2014). The DPPH radical oxidation of B1 and B2 has been found to result in the formation of A1 (EC-(4*β*→8,2*β*→*O*→7)-C) and A2 (EC-(4*β*→8,2*β*→*O*→7)-EC) (Kondo et al., 2000; Osman and Wong, 2007; Fischer et al., 2025). The use of copper (II) chloride as an oxidant agent showed that B3 and B4 form spiro-linked oxidation products, which were also found in red rice bran (Hayashi et al., 2018; Hibi and Yanase, 2019; Kataoka et al., 2024). When B2 is oxidised with copper (II) chloride in an aqueous solution, no A2 is produced, whereas the DPPH-induced oxidation produces A2 (Kondo et al., 2000; Suwa and Yanase, 2022).

The B-types can be obtained by chromatographic purification from natural sources such as grape seed, cacao bean, and apple (Köhler et al., 2008b; Appeldoorn et al., 2009; Li et al., 2010, 2016). To enhance their concentration, a semi-synthetic approach is described by Köhler et al. (2008a), that includes acid-catalysed degradation of enriched polymeric PCs and the addition of nucleophiles such as (+)-catechin and (−)-epicatechin yielding dimeric PCs. This method was frequently used to isolate dimeric B-type PCs from side streams of the agricultural industry, such as black chokeberry pomace (*Aronia melanocarpa*), grape seed (*Vitis vinifera*), and willow bark (*Salix alba*), as part of the zero-waste strategy (Bai et al., 2019, Esatbeyoglu et al., 2010, Esatbeyoglu and Winterhalter, 2010, Köhler et al., 2008a).

A deeper understanding of how B-type PCs are converted under oxidative conditions requires detailed structural characterisation of their oxidation products. In case of determining the interflavan bond, for example, long-range ^1^H–^13^C correlations, observable in the nuclear magnetic resonance (NMR) spectroscopy based ^1^H–^13^C HMBC experiment, are essential to verify the C4→C8 linkage of the units (Balas and Vercauteren, 1994; Esatbeyoglu et al., 2011). Circular dichroism (CD) spectroscopy is another essential tool for determining the absolute configuration of the interflavan bond. A study by Barrett et al. (1979) measured the CD-spectra of various A- and B-type PCs. This study showed that a negative Cotton effect at λ = 200–220 nm indicates an *α*-orientation (4S-configuration) of the interflavan bond, as observed in B3 and B4, while a positive Cotton effect at λ = 200–220 nm indicates a *β*-orientation (4R-configuration), as determined for the B1, B2, and A2 (Barrett et al., 1979). Ion mobility spectrometry (IMS) is an analytical tool to separate isomers and isobars with the same mass-to-charge ratio (*m*/*z*) according to size, shape, and charge (Eiceman and Karpas, 2005; Kanu et al., 2008; Lanucara et al., 2014). Further insights into the structural properties can be provided by the collision cross section (CCS) values, which are derived from the drift time due to the different effective cross-section of the molecule with the gas phase and are typically expressed in Å^2^ (Kanu et al., 2008; Lanucara et al., 2014). In trapped ion mobility spectrometry (TIMS), the generated ions are kept stationary (trapped) in a moving gas stream, which offers better resolution compared to classical ion mobility spectrometers (Ridgeway et al., 2018).

The interest in A- and B-types is driven by their correlation with various health-promoting properties, including antioxidant, anti-inflammatory, and antibacterial effects (Terra et al., 2011; Katoh et al., 2012; Wang et al., 2015). Because of the challenging isolation of these substances, only a limited number of studies have simultaneously used A- and B-type PAs to compare their potential health related benefits. A comparative study by Plumb et al. (1998) of the antioxidant activity of A- and B-type PCs, analysed using Trolox Equivalent Antioxidant Capacity (TEAC) assay, revealed that the B-types (B1–B4, B5, B7) had higher ABTS radical scavenging properties compared to the A-type A2. However, A2 and the different B-types did not show significant differences in the lipid peroxidation assay (Plumb et al., 1998). The study by Saint-Cricq De Gaulejac et al. (1999) confirmed the higher ability of B2 to scavenge superoxide anion radicals compared to A2. This is explained by the fact that A2 has one less hydroxyl group at position C7t due to the ether bridge between C2u–*O*–C7t (Saint-Cricq De Gaulejac et al., 1999). Dong et al. (2013) showed that, in an aqueous assay, the B-type demonstrated a higher hydroxyl radical and DPPH radical scavenging activity than the A-type. In contrast, in a lipid system, a comparable antioxidant activity or even a higher activity of the A-type compared to the B-type was observed (Dong et al., 2013).

The current study aimed to identify the primary oxidation products resulting from DPPH radical-induced oxidation of the most prevalent dimeric B-type PCs (B1–B4), given the limited availability or complete absence of reference substances. Following structure elucidation using low-temperature nuclear magnetic resonance (NMR) spectroscopy, mass spectrometry (MS), and circular dichroism (CD), the potential of ion mobility separation (TIMS-TOF) for rapid structure verification was investigated. A key objective was to determine the antioxidant activity of A- and B-type PCs, as well as spiro-linked PCs, which have been less explored, for the first time.

## 2 Materials and methods

### 2.1 Chemicals

The PURELAB® flex 3 (ELGA LabWater, Veolia Water Technologies, Celle, Germany) was used to prepare ultrapure water. For HPLC-PDA measurements, acetonitrile UHPLC supergradient (>99.9 %) from PanReac AppliChem ITW Reagents (Darmstadt, Germany) was acquired. For LR-HPLC-ESI-MS/MS measurements, acetonitrile LC-MS grade (>99.9 %) obtained from Honeywell Riedel-de Haën™ (Seelze, Germany) and acetic acid (LC-MS grade) from Honeywell Fluka™ (Seelze, Germany) were used. The UHPLC-TIMS-TOF analyses required water (LC-MS grade) and acetonitrile (UHPLC-MS grade) from Th. Geyer (Renningen, Germany) and formic acid (≥99.8 %, LC-MS grade) was obtained from Fisher Scientific (Loughborough, UK). Ethanol (99 %, denatured with 1 % methyl ethyl ketone) was ordered from Walter CMP (Kiel, Germany). Acetic acid (Rotipuran®, 100 %) and methanol (Rotisolv®, HPLC gradient ≥99.9 %) were acquired from Carl Roth (Karlsruhe, Germany). Hydrochloric acid conc. (37 %), ammonium acetate (p. a.) and potassium persulphate (p. a.) were ordered from Merck KGaA (Darmstadt, Germany). Sodium hydrogen carbonate (≥99.7 %), sodium acetate anhydrous (chemical pure), iron (III) chloride hexahydrate (≥99 %), and copper (II) chloride (water-free) were obtained from Chemsolute (Renningen, Germany). Neocuproine (≥98 %), (±)-6-hydroxy-2,5,7,8-tetramethylchroman-2-carboxylic acid (Trolox; 97 %), 2,4,6-tri-(2-pyridyl)-s-triazine (TPTZ; ≥98 %), 2,2′-azino-bis(3-ethylbenzothiazoline-6-sulfonic acid) diammonium salt (ABTS; ≥98 %), and quercetin hydrate (≥95 %) were purchased from Sigma Aldrich (Steinheim, Germany). 2,2-Diphenyl-1-picrylhydrazyl (DPPH, free radical with 95 %) was acquired from abcr (Karlsruhe, Germany). Acetone-*d*_6_ (purity 99.8 %) was ordered by Deutero (Kastellaun, Germany). Procyanidins A1 and A2 were purchased from Extrasynthese (Lyon, France).

### 2.2 Preparation of B1, B2, B3, and B4

*Aronia melanocarpa* pomace was used for the semi-synthesis of the dimers B1 (EC-(4*β*→8)-C) and B2 (EC-(4*β*→8)-EC) according to the method of Esatbeyoglu and Winterhalter (2010). *Salix alba* bark was used as raw material for the semi-synthetic preparation of B3 (C-(4*β*→8)-C) and B4 (C-(4*β*→8)-EC) following the method of Esatbeyoglu et al. (2010). The chromatographic purity of all B-types was above 90 % at λ = 280 nm. HPLC-photodiode array (PDA) and LC-ESI-MS/MS analysis verified all substances by comparison with previously isolated standards published in Esatbeyoglu et al. (2010) and Esatbeyoglu and Winterhalter (2010).

### 2.3 HPLC-PDA

HPLC-PDA analysis was performed on an Agilent Technologies (Waldbronn, Germany) 1290 series ultra-high-performance liquid chromatographic (UHPLC) equipped with a G7120A high-speed pump, a G7167B multisampler, and a G7117B PDA detector. The HPLC conditions are described in Fischer et al. (2025).

### 2.4 HPLC-low-resolution (LR)-ESI-MS/MS

HPLC-ESI-MS/MS analysis was performed on an Agilent 1100 series HPLC equipped with a G1312A binary pump, a G1329B autosampler, and a G1315B PDA detector. HCT-ultra ETD II (ESI-Ion Trap MS/MS) from Bruker Daltonics (Bremen, Germany) for mass spectrometric (MS) analysis was coupled with the HPLC system. LC and MS conditions are described in previous work (Fischer et al., 2025).

### 2.5 UHPLC trapped ion mobility spectrometry (TIMS)-Time of flight (TOF) mass spectrometry

TIMS-TOF-MS spectra were acquired on the following system: Agilent Infinity 1290 series UHPLC consisting of a binary pump (G4220A), an autosampler (G4226A), and a PDA-detector (G4212A). The separation was carried out on an Agilent ZORBAX Eclipse Plus C18 column (2.1 × 100 mm, 1.8 μm, 95 Å) with a column temperature of 30 °C. As a mobile system, water acidified with 0.1 % formic acid (A) and acetonitrile acidified with 0.1 % formic acid (B) were used. The following gradient with a flow rate of 0.2 mL/min was applied: 2–20 % B (0–25 min), 20–35 % B (25–35 min), 35–98 % B (35–40 min), and 98 % B (40–43 min) with a re-equilibration step of 5 min. The UHPLC was connected to a trapped ion mobility spectrometry (TIMS) and electrospray ionisation (ESI) time-of-flight-mass spectrometry (TOF) from Bruker Daltonics. The settings for the ESI-MS/MS measurements were as follows: Positive = scan range, *m*/*z* = 20–1350; inverse ion mobility range, 1/*K*o *=* 0.55–1.90 V∗s∗cm^−2^; ramp time = 100 ms; capillary voltage = 4500 V; nebulising gas pressure = 2.2 bar (N_2_); dry gas flow rate = 10 L/min (N_2_); nebuliser temperature = 220 °C, and collision energy = 10 eV. Negative = scan range, *m*/*z* = 20–1300; inverse ion mobility range, 1/*K*_*o*_ = 0.45–1.45 V∗s∗cm^2^; ramp time = 100 ms; capillary voltage = 3600 V; nebulising gas pressure = 2.20 bar (N_2_); dry gas flow rate = 10 L/min (N_2_); nebuliser temperature = 220 °C, and collision energy = 10 eV. The ESI-L Low Concentration Tuning Mix from Agilent Technologies was used to calibrate the MS and TIMS. To operate the system, the software Bruker Compass Hystar version 6.2 and otofControl version 6.2 (Bruker Daltonics) were used. For evaluating the analyses, Bruker Compass Data Analysis version 5.3 (Bruker Daltonics) was used.

### 2.6 Nuclear magnetic resonance spectroscopy (NMR)

^1^H, ^13^C, and 2D NMR spectroscopy, including ^1^H–^1^H COSY, multiplicity-edited ^1^H–^13^C HSQC, ^1^H–^13^C HMBC, ^1^H–^1^H TOCSY, and ^1^H–^1^H ROESY NMR experiments, were performed on an Bruker Avance NEO spectrometer operating at 14.1 T (*ν*_L_ (^1^H) = 600.33 MHz, *ν*_L_ (^13^C) = 150.95 MHz) equipped with a DUL cryo-probe (Bruker BioSpin, Rheinstetten, Germany). All measurements were performed at 253.0 K. Additional ^1^H NMR spectra were measured at 298 K. All samples were dissolved in acetone-*d*_*6*_ and transferred to 5 mm Boro 3.3 NMR tubes (Deutero). The coupling constants (*J*) are given in Hertz (Hz), and the chemical shift (δ) in parts per million (ppm) relative to the residual resonances of the solvent. All spectra were referenced to the residual solvent resonances of acetone-*d*_*6*_ (δ_H_ = 2.05 ppm and δ_C_ = 206.3 ppm, Gottlieb et al., 1997). For the ^1^H–^1^H ROESY NMR spectrum a spinlock time of 175 ms was applied. The NMR spectra were processed and analysed with TopSpin version 4.3.0 (Bruker BioSpin) and MestReNova version 14.2.2 (Mestrelab Research, Santiago de Compostela, Spain).

### 2.7 Circular dichroism (CD)

A Jasco J-815 CD spectrophotometer (Gross-Umstadt, Germany) was used for the measurement of the CD spectra, with the following parameters: Wavelength λ = 200–350 nm, bandwidth = 1.00 nm, scanning speed = 50.0 nm/min, data interval = 0.10 nm and temperature = 20.0 °C. For the spectral analysis, 0.4 mmol/L solutions were prepared with methanol (gradient grade) and measured in a Hellma 110-QS cuvette (Müllheim, Germany) with a 1 mm path length. Molar ellipticity [θ_λ_] was calculated according to equation (1) with *θ*_*λ*_ as measured ellipticity (deg), *c* as molar concentration (mol/L), and *l* as layer thickness (cm):

(1) $$\left[\theta_{\lambda }\right]=\left(\theta_{\lambda \;}x\;100\right)/\left(c\;x\;l\right)$$

### 2.8 Geometry optimisation and predicted circular dichroism (CD)

The geometry optimisation was performed using density functional theory (DFT) with the B3LYP functional (Becke, 1993) and the 6-31G(d) basis set (Del Bene et al., 1995). Interactions with the solvent acetone were included using the Conductor-like Polarizable Continuum Model (CPCM) (Barone and Cossi, 1998) employing ORCA software version 6.0.1 (Neese, 2012, 2022). The optimised structure was visualised with Avogadro 2, an open-source molecular builder and visualisation tool version 1.100 (http://two.avogadro.cc/) (Hanwell et al., 2012). The CD spectra were obtained from time-dependent DFT (TD-DFT) calculations using the B3LYP functional and def2-SVP basis set (Weigend and Ahlrichs, 2005) with CPCM of methanol for geometry optimisation and followed by B3LYP/def2-TZVP (Weigend and Ahlrichs, 2005) calculations with 25 roots (excitations) for the predicted CD spectrum by using ORCA. For all optimisations, a frequency calculation was carried out using the same theory to confirm no imaginary frequency. Subsequently, the CD spectrum was generated by the ORCA TD-DFT data set using ChimeraX version 1.9 (Meng et al., 2023) and the SEQCROW plugin version 1.8.18 (Schaefer et al., 2021; Ingman et al., 2021).

### 2.9 DPPH radical-induced oxidation of B-type procyanidins and isolation of their oxidation products

DPPH radical-induced oxidation was carried out under the optimal reaction conditions for the maximum formation of the main oxidation products of the individual B-type PCs (B1–B4), according to Fischer et al. (2025). Table 1 lists the applied reaction conditions.

**Table 1:** Optimal reaction conditions for the preparative scale preparation of the main oxidation products of the B-type dimers B1–B4 according to Fischer et al. (2025).

B-type	Oxidation product	Time [min]	Temperature [°C]	Ratio B-type to DPPH radical (*n*/*n*)
B1	A1 (1)	77.2	61.4	9:28.8
B2	A2 (2)	10.0	66.9	9:26.5
B3	Oxidation product 1 of B3 (3)	10.0	25.0	9:50
B3	Oxidation product 2 of B3 (4)	314	75.0	9:12.5
B4	Oxidation product 2 of B4 (5)	360.0	53.7	9:12.5

The reactions were conducted in batches of 1 L in a three-necked flask. Depending on the B-type, 900 mL of 0.10 mM aqueous B-type solution was combined with a mixture of 100 mL of methanol and the corresponding volumes of 5 mM methanolic DPPH radical solution to obtain the correct ratio of B-type to DPPH radical. At the end of the reaction time, the solution was cooled in a cooling bath at −50 °C to slow down the reaction. Then, the solution was filtered through a Nalgene™ Rapid-Flow™ 0.2 μm PES filter (Thermo Fischer Scientific, Waltham, Massachusetts, USA). Methanol was removed under reduced pressure at 30 °C, and the solution was lyophilised (alpha 3–4 LSCplus, Martin Christ Gefriertrocknungsanlagen, Osterode am Harz, Germany).

The purification of the oxidation products and the B-type dimers B1–B4 were carried out on a preparative HPLC from ECOM (Chrášťany u Prahy, Czech Republic), equipped with a preparative pump (ECP 2050), a four-way gradient valve (ECB 2007), a UV/Vis-detector at λ = 280 nm (TOY20DAD 800 H), and a 2-channel online degasser (A5328) from Knauer (Berlin, Germany) under the same parameters as described in Fischer et al. (2025). The products were obtained and characterised as listed below:

*Procyanidin A*1 *(1):* off-white solid; ESI-MS: *m*/*z* 575.1198 [M−H]^−^ (calculated for C_30_H_24_O_12_: 575.1195 [M−H]^−^, Table 2); CD (0.4 mmol/L in methanol) [θ]_211_ = +45,732, [θ]_238_ = +23,593, [θ]_272_ = −16,407; ^1^H and ^13^C NMR data in Table 3; CCS value: 235.6 Å^2^; yield: 36.7 %

**Table 2:** UHPLC-ESI-TIMS-TOF-MS analysis of the isolated B-type dimeric procyanidins B1–B4 and the oxidation (Ox.) products 1–5.

Compound	*m*/*z* [M+H]^+^ experimental	Formula	Mass error [ppm]	Predicted CCS^a^ [Å^2^]	CCS [Å^2^] [M+H]^+^	Mobility [1/*K*_*o*_]
B1	579.1485	C_30_H_26_O_12_	2.0	233.2	227.6	1.110
B2	579.1485	C_30_H_26_O_12_	1.9	233.2	226.4	1.104
B3	579.1488	C_30_H_26_O_12_	1.5	233.2	224.3	1.094
B4	579.1495	C_30_H_26_O_12_	0.4	233.2	216.4	1.055
A1 (1)	577.1339	C_30_H_24_O_12_	0.3	228.3	235.7	1.149
A2 (2)	577.1338	C_30_H_24_O_12_	0.5	228.3	235.9	1.151
Ox. product 1 of B3 (3)	577.1342	C_30_H_24_O_12_	−0.2	234.8	220.2	1.074
Ox. product 2 of B3 (4)	577.1342	C_30_H_24_O_12_	−0.3	234.8	219.8	1.072
Ox. Product 2 of B4 (5)	577.1342	C_30_H_24_O_12_	−0.3	234.8	220.3	1.059

a Predicted by https://ccsbase.net (Ross et al., 2020).

**Table 3:** ^1^H NMR (ν_L_ = 600.33 MHz) and ^13^C NMR (ν_L_ = 150.95 MHz) data of the A-type dimeric procyanidins A1 (1) and A2 (2) at 253.0 K.

Ring	No.	Procyanidin A1 (1)		Procyanidin A2 (2)	
		δ_C_ [ppm], type	δ_H_ [ppm], multiplicity *J*_HH_ [Hz]	δ_C_ [ppm], type	δ_H_ [ppm], multiplicity *J*_HH_ [Hz]
C	2u	98.9, C		98.8, C	
	3u	66.2, CH, C3u–OH	4.11, dd (4.50, 3.43) 4.69, d (4.50)	66.4, CH C3u–OH	4.12, dd (4.60, 3.34) 4.70, d (4.60)
	4u	27.7, CH	4.16, d (3.43)	27.6, CH	4.28, d (3.34)
A	4au	102.7, C		102.9, C	
	5u	155.5, C, C5u–OH	7.09, s	155.8, C, C5u–OH	7.39, s
	6u	96.7, CH	5.94, d (2.35)	96.9, CH	5.95, d (2.36)
	7u	157.0, C, C7u–OH	8.92, s	157.0, C, C7u–OH	8.90, s
	8u	95.1, CH	6.04, d (2.35)	95.1, CH	6.04, d (2.36)
	8au	152.9, C		152.9, C	
B	1u′	130.9, C		131.1, C	
	2u′	114.4, CH	7.13, d (2.16)	114.4, CH	7.14, d (2.16)
	3u′	145.3, C C3u′–OH	8.55, s	145.3, C C3u′–OH	8.54, s
	4u′	144.2, C C4u′–OH	8.70, s	144.2, C C4u′–OH	8.69, s
	5u′	114.1, CH	6.81, d (8.29)	114.1, CH	6.82, d (8.28)
	6u′	118.5, CH	7.00, dd (8.29, 2.16)	118.5, CH	7.01, dd (8.28, 2.16)
F	2t	83.7, CH	4.61, d (8.86)	80.6, CH	4.94, d (1.35)
	3t	66.4, CH C3t–OH	4.14, dddd (9.40, 8.86, 5.93, 4.79) 4.63, d (4.79)	64.9, CH C3t–OH	4.28, m^a^ 4.44, d (5.45)
	4t	29.0, CH_2_	2.53, dd (16.37, 9.40) 3.02, dd (16.37,5.93)	29.8, CH_2_	2.78, ddd (17.02, 1.72, 1.35) 2.91, dd (17.02, 4.62)
D	4 at	102.2, C		101.2, C	
	5t	154.7, C C5t–OH	9.41, s	155.3, C C5t–OH	9.29, s
	6t	95.3, CH	6.13, s	95.1, CH	6.12, s
	7t	150.8, C		150.8, C	
	8t	105.3, C		105.3, C	
	8 at	150.2, C		150.7, C	
E	1t′	128.8, C		129.8, C	
	2t′	115.1, CH	7.01, d (1.80)	115.2, CH	7.29, d (2.07)
	3t′	145.5, C C3t′–OH	8.69, s	145.2, C C3t′–OH	8.56, s
	4t′	144.9, C C4t′–OH	8.73, s	144.7, C C4t′–OH	8.65, s
	5t′	114.8, CH	6.83, d (8.11)	114.5, CH	6.84, d (8.18)
	6t′	119.9, CH	6.86, dd (8.11, 1.80)	119.5, CH	7.04, dd (8.18, 2.07)

a Due to signal overlap, the expected ddd (*J*_HH_ = 4.62, 1.72, 1.35 Hz) coupling pattern based on *J*_HH_ couplings detected in neighbouring H4t and H2t protons could not be observed.

*Procyanidin A2 (2):* off-white solid; ESI-MS: *m*/*z* 575.1194 [M−H]^−^ (calculated for C_30_H_24_O_12_: 575.1195 [M−H]^−^, Table 2); CD (0.4 mmol/L in methanol) [θ]_211_ = +87,648, [θ]_238_ = +48,783, [θ]_211_ = −16,394; ^1^H and ^13^C NMR data in Table 3; CCS value: 236.1 Å^2^; yield: 18.4 %

*Oxidation product 1 of B3 (3):* white solid; ESI-MS: *m*/*z* 575.1190 [M−H]^−^ (calculated for C_30_H_24_O_12_: 575.1195 [M−H]^−^, Table 2); CD (0.4 mmol/L in methanol) [θ]_211_ = +212,078, [θ]_238_ = +50,043, [θ]_294_ = −27,629; ^1^H and ^13^C NMR data in Table 4; CCS value: 220.1 Å^2^; yield:11.8 %

**Table 4:** ^1^H NMR (ν_L_ = 600.33 MHz) and ^13^C NMR (ν_L_ = 600.95 MHz) data of oxidation products 1 (3) and 2 (4) of B3 as well as oxidation products of B4 (5).

	No.	Oxidation product 1 of B3 (3)		Oxidation product 2 of B3 (4)		Oxidation product of B4 (5)	
		δ_C_ [ppm], type	δ_H_ [ppm], multiplicity *J*_HH_ [Hz]	δ_C_ [ppm], type	δ_H_ [ppm], multiplicity *J*_HH_ [Hz]	δ_C_ [ppm], type	δ_H_ [ppm], multiplicity *J*_HH_ [Hz]
C	2u	74.2, CH	4.87, dd (2.72, 1.92)	74.1, CH	4.88, dd (2.65, 1.80)	74.0, CH	4.82, dd (2.78, 1.86)
	3u	60.9, CH C3u–OH	4.05, ddd (4.60, 3.03, 2.72) 4.37, d (4.60)	61.2, CH C3u–OH	4.05, ddd (4.98, 2.77, 2.65) 4.34, d (4.98)	61.1, CH C3u–OH	4.01, ddd (4.94, 2.89, 2.78) 4.40, d (4.94)
	4u	37.5, CH	4.02, dd (3.03, 1.92)	38.0, CH	4.03, dd (2.77, 1.80)	37.6, CH	4.00, dd (2.89, 1.86)
A	4au	99.1, C		98.7, C		98.7, C	
	5u	154.7, C C5u–OH	8.69, s	154.0, C C5u–OH	8.61, s	153.9, C C5u–OH	8.66, s
	6u	95.0, CH	5.77, d (2.30)	94.9, CH	5.56, d (2.30)	94.9, CH	5.50, d (2.27)
	7u	157.6/158.0, C C7u–OH	8.64, s	157.6/157.6, C C7u–OH	–	157.6, C C7u–OH	–
	8u	95.2, CH	6.08, d (2.30)	94.46, CH	5.85, d (2.30)	94.7, CH	6.04, d (2.27)
	8au	157.6/158.0, C		157.6/157.6, C		157.4, C	
B	1u′	129.2, C		129.1, C		129.2, C	
	2u′	116.3, CH	6.77, s	116.5, CH	6.86, s	116.6, CH	6.77, s
	3u′	144.7, C C3u′–OH	8.37, s	145.8, C C3u′–OH	–	145.9, C C3u′–OH	–
	4u′	145.9, C C4u′–OH	8.09, s	144.9, C	–	144.6, C C4u′–OH	–
	5u′	116.0, CH	6.23, s	116.5, CH	6.53, s	115.9, CH	6.46, s
	6u′	125.2, C		124.8, C		124.6, C	
F	2t	84.4, CH	4.16, d (9.38)	81.3, CH	4.74, d (1.28)	84.6, CH	4.22, d (9.45)
	3t	66.9, CH C3t–OH	3.71, dddd (9.94, 9.38, 6.01, 4.97) 4.27, d (4.97)	64.2, CH C3t–OH	4.17, ddd 2.30, 3.75, 3.80 3.87, d (3.75)	66.7, CH C3t–OH	3.83, dddd (10.25, 9.45, 5.51, 4.86) 4.32, d (4.86)
	4t	29.9, CH_2_	2.22, dd (16.44, 9.94) 2.84, dd (16.44, 6.01)	28.5, CH_2_	2.46, dd (16.39, 3.80) 2.69, ddd (16.39, 2.30, 1.28)	29.3, CH_2_	2.16, dd (16.05, 10.25) 2.92, dd (16.05, 5.51)
D	4 at	115.1, C		112.5, C		114.0, C	
	5t	189.8, C		189.6, C		189.1, C	
	6t	50.2, CH_2_	3.30, d (19.08) 4.33, d (19.08)	51.6, CH_2_	3.26, d (18.05) 4.46, d (18.05)	51.1, CH_2_	3.26, d (18.30) 4.44, d (18.30)
	7t	203.5, C		203.9, C		203.9, C	
	8t	68.8, C		68.6, C		68.3, C	
	8 at	170.2, C		170.6, C		170.4, C	
E	1t′	128.9, C		129.9, C		129.5, C	
	2t′	115.6, CH	6.31, d (2.02)	114.0	6.51, d (2.00)	115.5	6.52, d (1.98)
	3t′	145.0, C C3t′–OH	7.73, s	144.4, C C3t′–OH	–	145.1, C C3t′–OH	–
	4t′	143.7, C C4t′–OH	7.94, s	144.3, C C4t′–OH	–	144.8, C C4t′–OH	–
	5t′	115.2, CH	6.47, d (8.25)	114.5, CH	6.42, d (8.17)	114.7, CH	6.47, d (8.15)
	6t′	119.5, CH	5.91, dd (8.25, 2.02)	118.8, CH	6.14, dd (8.17, 2.00)	120.2, CH	6.31, dd (8.15, 1.98)

*Oxidation product 2 of B3 (4):* white solid; ESI-MS: *m*/*z* 575.1194 [M−H]^−^ (calculated for C_30_H_24_O_12_: 575.1195 [M−H]^−^, Table 2); CD (0.4 mmol/L in methanol) [θ]_210_ = +204,341, [θ]_239_ = +27,489, [θ]_289_ = −47,937; ^1^H and ^13^C NMR data in Table 4; CCS value: 219.9 Å^2^; yield: 3.43 %

*Oxidation product of B4 (5):* white solid; ESI-MS: *m*/*z* 575.1190 [M−H]^−^ (calculated for C_30_H_24_O_12_: 575.1195 [M−H]^−^, Table 2); CD (0.4 mmol/L in methanol) [θ]_212_ = +133,096, [θ]_231_ = +40,760, [θ]_290_ = −3446; ^1^H and ^13^C NMR data in Table 4; CCS value: 219.9 Å^2^; yield: 15.1 %

### 2.10 Antioxidant activity

The determination of the antioxidant activity was performed using two photometric assays (DPPH- and TEAC-assay) investigating the radical scavenging properties and two assays analysing the reducing properties of a copper (II) neocuproine (Cupric Ion Reducing Antioxidant assay; CUPRAC) and a Fe(III)-TPTZ complex (Ferric Reducing Antioxidant Power assay; FRAP). The absorption was measured with the multimode plate reader TECAN infinite M200 (Männedorf, Switzerland). All assays were performed in three replications.

All results were expressed as the ratio between the measured antioxidant activity as Trolox equivalents (TE) and the molar concentration of the tested compound. The antioxidant activity was determined using an external Trolox calibration curve to calculate the molar concentration as TE, and the resulting TE molar concentration was divided by the molar concentration of the compound used in the assay (equation (2).

(2) $$\text{Antioxidant}\;\text{activity}\;\left(\frac{TE\;mol/L\;}{mol/L}\right)=\frac{\;\text{molar}\;\text{concentration}\;\text{TE}\;\left(mol/L\right)\;}{\text{molar}\;\text{concentration}\;\text{of}\;\text{substance}\;\left(mol/L\right)\;}$$

#### 2.10.1 2,2-Diphenyl-1-picrylhydrazyl (DPPH) assay

The DPPH assay was adapted from Brand-Williams et al. (1995) with small modifications. In a 96-well microtiter plate, 100 μL of a 300 μM ethanolic DPPH radical solution was added, along with 100 μL of an ethanolic blank solution/positive control (quercetin, 66 μM)/calibration standards (5–120 μM Trolox), as well as the sample (35–70 μM). After an incubation time of 30 min in the dark at room temperature, the absorption was measured at λ = 515 nm.

#### 2.10.2 Trolox Equivalent Antioxidant Capacity (TEAC) assay

The TEAC assay was performed based on Arts et al. (2004) with modifications. An aqueous solution of 7 mM ABTS and 2.5 mM potassium persulfate was kept in the dark at room temperature for 12 h. Then, the solution was diluted with ethanol to an absorption of 0.7 ± 0.02 at λ = 734 nm. From this solution, 200 μL and 10 μL of each blank (ethanol)/sample (90–350 μM)/positive control (quercetin, 170 μM), and calibration standards of Trolox (0.1–1.0 mM) were added to a 96-well microtiter plate. After 6 min, the absorption was measured at λ = 734 nm.

#### 2.10.3 Cupric Ion Reducing Antioxidant capacity (CUPRAC) assay

The CUPRAC assay was carried out with modifications according to Apak et al. (2004). An ethanolic quercetin solution (500 μM) was used as a positive control. First, 5 μL of the blank (ethanol)/positive control/sample (260–500 μM), and calibration standards (0.2–4 mM Trolox) were pipetted to a 96-well microtiter plate. Afterwards, 200 μL of a reaction solution with 50 μL copper (II) chloride (10 mM), 50 μL ethanolic neocuproine (7.5 mM), 50 μL ammonium acetate buffer (1 M, pH 7), and 50 μL water were added. Subsequently, the 96-well microtiter plate was incubated for 30 min in the dark, and the absorbance was measured at λ = 450 nm.

#### 2.10.4 Ferric Reducing Antioxidant Power (FRAP) assay

The FRAP assay was carried out according to Benzie and Strain (1996). Here, 5 μL of the blank (ethanol)/positive control (quercetin, 230 μM)/sample (120–485 μM), and calibration standards of Trolox (analogue to TEAC assay), as well as 97.5 μL water, 25 μL sodium acetate buffer (10 mM) and 45 μL FRAP reagent were added to a 96-well microtiter plate. The FRAP reagent was prepared by adding equal volumes of 1 mL iron (III) chloride aqueous solution (20 mM), 1 mL 10 mM TPTZ solution in 20 mM aqueous HCl, and 1 mL 10 mM sodium acetate buffer. The plate was incubated in the dark at 40 °C, and the absorption was measured at λ = 595 nm.

### 2.11 Statistical analysis

The statistical analysis of the different antioxidant activity assays was performed using GraphPad Prism version 10.2.1 (Boston, Massachusetts, USA). The normality of the distribution was assessed using the Shapiro-Wilk test, followed by a one-way ANOVA with Tukey's multiple comparison test. Differences were significant at *p <* 0.05, and data are presented as means ± SD (standard deviation).

## 3 Results and discussion

### 3.1 Oxidation products of B1 and B2

**Fig. 1:**
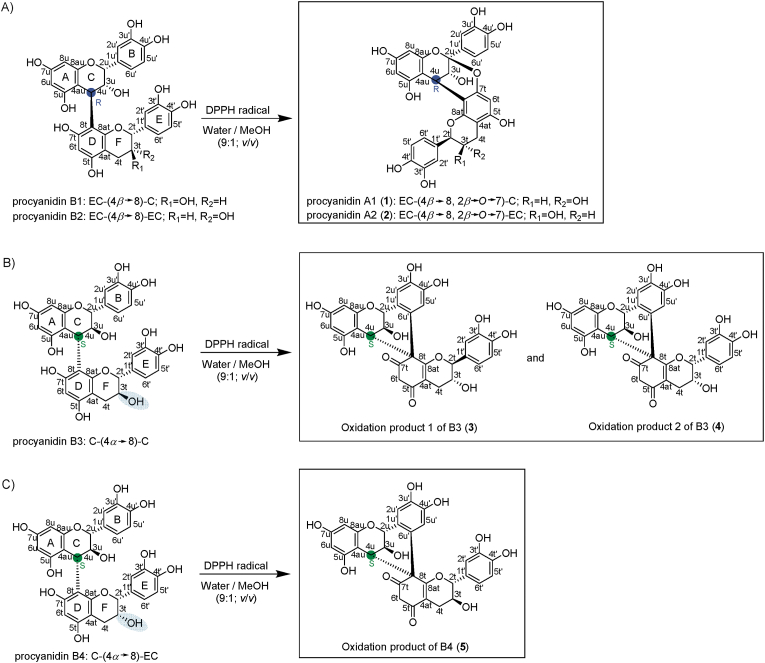
DPPH radical-induced oxidation of dimeric B-types: A) B1 and B2 formed the corresponding A-types (1, 2), B) B3 formed the spiro-linked oxidation products (3, 4), and C) B4 formed spiro-linked product 5. The important stereogenic centres are highlighted.

In our previous study, empirical models for the formation of the main oxidation products were obtained for all C4→C8-linked dimers using the design of experiment (DoE) to obtain the optimal formation rates (Fischer et al., 2025). These conditions were scaled up from an analytical to a preparative scale. The preparative scale conversion of B1 (EC-(4*β*→8)-C) showed a low formation of by-products (Figure S1 A, supplementary material), demonstrating the selectivity of the conversion under the selected reaction parameters, even on a preparative scale. The percentage formation of the targeted oxidation product of B1 (1) (*m*/*z* 575 [M−H]^−^), known as A-type A1, was approx. 73 % (determined at λ = 280 nm). Following oxidation, less than 2 % B1 remained. After purification by preparative HPLC, PC A1 (1) (Fig. 1 A) was obtained with a chromatographic purity of 99.3 %, determined chromatographically at λ = 280 nm, and a total yield of 36.7 %.

The oxidation of B2 (EC-(4*β*→8)-EC) to A2 (2) showed an increased formation of by-products by LC-MS-PDA analysis (Figure S1 B). The oxidation of B2 led to the formation of approx. 28 % A2 (2; purity = 98.4 % at λ = 280 nm, yield 18.4 %), while about 20 % of unreacted B2 remained. This unreacted B2 can be recovered through purification by preparative HPLC. Furthermore, substances with *m*/*z* 573 [M−H]^−^ were formed during the reaction (Figure S1 B). These compounds may be analogous to those observed in the study of Suwa and Yanase (2022), where various oxidation products (*m*/*z* 573 [M−H]^−^) with a macrocyclic ether structure were generated by the copper (II) chloride-induced oxidation of B2. However, it is also possible that these compounds contain two ether bridges (Poupard et al., 2011; De Taeye et al., 2017).

The oxidation of polyphenols by the DPPH radical can occur via hydrogen atom transfer (HAT), single-electron transfer-proton transfer (SET-PT), or sequential proton loss electron transfer (SPLET) (Gulcin and Alwasel, 2023). Kondo et al. (2000) demonstrated that the oxidation of the dimers B1 and B2 by the DPPH radical resulted in the formation of the corresponding A-type compounds A1 and A2. This process involves the abstraction of the C2 hydrogen by the DPPH radical, forming a quinone methide intermediate. Subsequently, an intermolecular rearrangement occurs, resulting in the formation of an ether bridge between C2u and C7t. During this process, the DPPH radical (violet) is reduced to DPPH-H (yellow) (Gulcin and Alwasel, 2023). This mechanism was also confirmed by Osman and Wong (2007) as well as Chen et al. (2014). An initial identification of compound 1 (A1) and 2 (A2) was carried out by reference samples. These references were used for retention time (R_t_) comparison (compound 1 and A1, R_t_ = 15.2 min; compound 2 and A2, R_t_ = 16.1 min). The TIMS-TOF-MS spectra of compounds 1 and 2 (A1 and A2) showed *m*/*z* values of 577.1339 [M+H]^+^/575.11198 [M−H]^−^ (1) and m/z 577.1338 [M+H]^+^/575.1194 [M−H]^−^ (2), indicating the molecular formula of C_30_H_24_O_12_ (calcd. *m*/*z* 577.1341 [M+H]^+^ and 575.1195 [M−H]^−^) for both compounds (Table 2). The fragmentation pattern of 1 and 2 were the same with *m*/*z* 557 [M−H−18 Da]^−^ (water lost), *m*/*z* 449 [M−H−126 Da]^−^ (retro Diels-Alder (RDA) product), *m*/*z* 423 [M−H−152 Da]^−^ (heterocyclic ring cleavage (HRF)), *m*/*z* 289 [M−H−286 Da]^−^ (quinone methide (QM) cleavage) and *m*/*z* 285 [M−H−290 Da]^−^ (QM cleavage) (Poupard et al., 2011; Rue et al., 2018). The comparison of retention time, fragmentation patterns, and NMR data confirmed the formation of A1 (1) and A2 (2) (Table 3).

The ^1^H and ^13^C NMR resonances of compounds 1 (A1) and 2 (A2) are shown in Table 3 (Fig. S2–5).

**Fig. 2:**
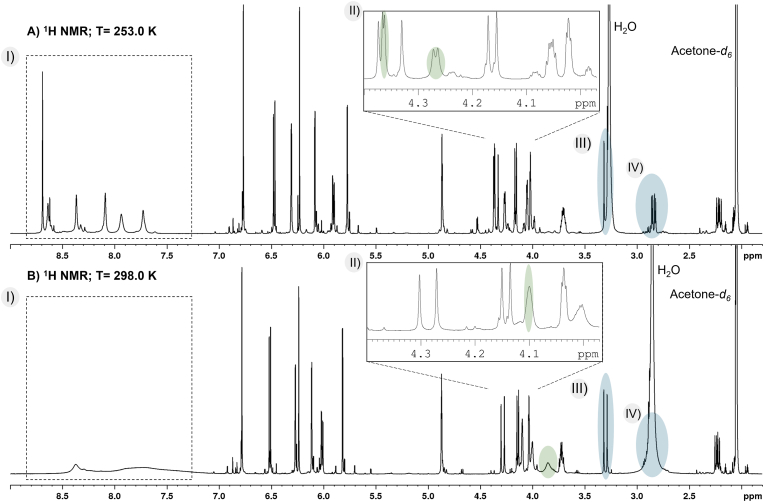
Temperature effect on the ^1^H NMR spectrum in acetone*‑d*_6_ from 3 A) at 253.0 K and B) at 298.0 K. The temperature induced changes of the aromatic hydroxyl protons (I), aliphatic hydroxyl protons (II), and the H4t and H6t proton signals (III and IV) are highlighted.

At 298.0 K, the aliphatic hydroxyl groups (C3t–OH and C3u–OH) showed broad singlet resonances due to the chemical exchange of the labile protons. In addition, the hydroxyl proton signals are in the range of the aromatic C-ring protons (Fig. 2 B, II). To slow down the chemical exchange and thus increase the resolution, the temperature for the NMR experiments was reduced to 253.0 K. This allowed the identification of the protons of the aromatic hydroxyl groups from A1 (1) and A2 (2). However, this was overall not possible for compounds 3–5 under the same conditions. Nevertheless, the low-temperature NMR measurements resulted in a sharpening of the detected resonances (Fig. 2 A–I). At 253.0 K, the protons of the hydroxyl groups C3t–OH and C3u–OH were observed as a doublet instead of a broad singlet due to coupling with the neighbouring protons (H3t/u) (Fig. 2 A, II). This phenomenon was only detectable at low-temperature and observed for all oxidation products (1–5). In addition, the lower temperature resulted in a deshielding of the water signal (Δδ = 0.41 ppm), which allowed an overall improved detection of the H4t (1–5) and H6t (3–5) resonances (Fig. 2 A, III–IV). For all these reasons, low-temperature NMR measurements are crucial for an accurate structure elucidation of the A-types (1, 2) and the oxidation products of B3 and B4 (3–5) (section 3.2).

An essential element for identifying PCs are the resonances from the ABX-system. For compound 1 these were the following resonances: δ_H_ = 7.13 ppm (d, *J*_HH_ = 2.16 Hz, 2u′), δ_H_ = 6.81 ppm (d, *J*_HH_ = 8.29 Hz, 5u′), δ_H_ = 7.00 ppm (dd, *J*_HH_ = 8.29, 2.16 Hz, 6u′), and δ_H_ = 7.01 ppm (d, *J*_HH_ = 1.80 Hz, 2t′), 6.83 ppm (d, *J*_HH_ = 8.11 Hz, 5t′), 7.86 ppm (dd, *J*_HH_ = 8.11, 1.80 Hz, 6t′), and δ_C_ at approx. 115 ppm for C2′, C5′, and C6′ indicating a catechol-ring with a dihydroxylation in *ortho*-position for the upper (u) and the terminal unit (t) (Kolodziej et al., 1991; Prasad, 2000). This was also the case for compound 2 (Table 3; Figure S2 and S4 ^1^H NMR spectra).

The A-type PCs with an ether bridge between C2u and O7t were characterised by their chemical shift of C2u at approx. δ_C_ = 100 ppm (δ_C_ = 98.9 ppm (1), δ_C_ = 98.8 ppm (2)) and the less shielded resonance for C7t at δ_C_ = 150 ppm (δ_C_ = 150.8 ppm (1), δ_C_ = 150.8 ppm (2)) due to the oxygen of the ether group (Kolodziej et al., 1991; Prasad, 2000). The main

**Fig. 3:**
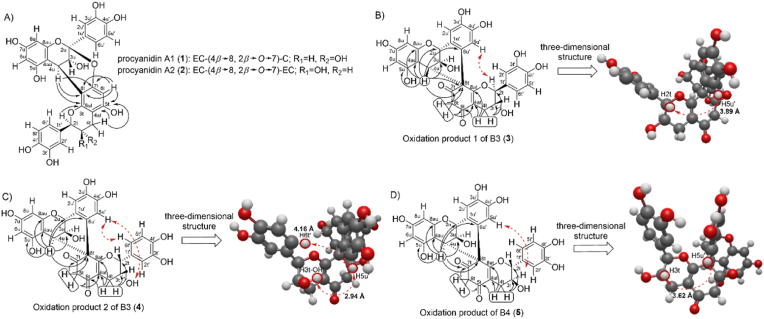
A) Important ^1^H–^13^C HMBC correlations indicated by solid arrows (→) of the A-types A1 (1) and A2 (2), B) oxidation product 1 of B3 (3), C) product 2 of B3 (4), and D) product of B4 (5) to characterise the new C4u–C8t–C6u′ linkage and the position of the carbonyl carbons, and the structurally significant ^1^H–^1^H ROESY correlations indicated by dashed arrows () on the three-dimensional structure of compound 3 to 5 optimised by B3LYP/6-31G(d) including the solvent interaction of acetone using the Conductor-like Polarizable Continuum Model (CPCM).

^1^H–^13^C HMBC correlations for the identification of the existing C4→C8 interflavan bond of compounds 1 and 2 are illustrated in Fig. 3 A.

The isolated compounds 1 and 2 differ only in the coupling constant of H2t with ^3^*J*_HH_ = 8.86 Hz (1) and ^3^*J*_HH_ = 1.35 Hz (2). From the Karplus curve, it can be concluded that in compound 1, the hydrogens H2t and H3t are in anti-periplanar conformation (^3^*J*_HH_ = 8.86 Hz; H (ax)–C2t–C3t–H (ax)), indicating a *trans*-configuration, which identifies compound 1 as A1. Compound 2 can be identified as PC A2 since H2t has a coupling constant of ^3^*J*_HH_ = 1.35 Hz. This indicated a *cis*-configuration of H (ax)–C2t–C3t–H (eq) (Karplus, 1960; Esatbeyoglu et al., 2013).

**Fig. 4:**
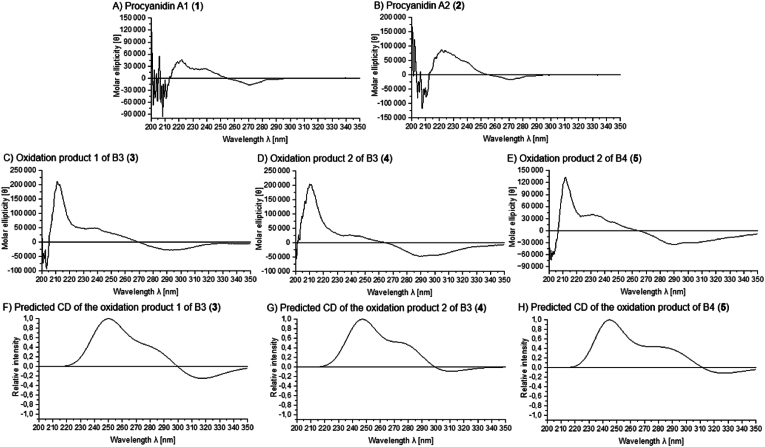
CD spectra between λ = 200–350 nm in methanol of A) procyanidin A1 (1), B) procyanidin A2 (2), C) oxidation product 1 of B3 (3), D) oxidation product 2 of B3 (4), and E) oxidation product of B4 (5), as well as F) the predicted spectrum of 3, G) the predicted spectrum of 4, and F) the predicted spectrum of 5 (optimisation were calculated by B3LYP/def2-SVP incorporating CPCM (methanol) followed by B3LYP/def2-TZVP with CPCM (methanol) for CD calculation).

In addition to NMR analysis, CD spectroscopy was used to verify the absolute stereochemistry of the interflavan bond. The CD spectra of A1 (1, [θ]_211_ = +45,732 with a shoulder at [θ]_238_ = +23,593) and A2 (2, [θ]_211_ = +87,648 with a shoulder at [θ]_238_ = +48,783) showed a positive Cotton effect for both compounds for the ^1^L_a_ band between λ = 200–220 nm (Fig. 4 A and B). These results indicated a *β-*orientation (4R-stereochemistry) of the interflavan bond, as expected for A1 and A2 (Barrett et al., 1979; Slade et al., 2005; Dudek et al., 2017). The measured CD spectra of A1 and A2 are consistent with those reported in previous literature. Lou et al. (1999) demonstrated that A1 and A2 both exhibit a positive Cotton effect for PCs at approx. λ = 220 nm, with a shoulder around λ = 240 nm. Barrett et al. (1979) also reported these spectral characteristics for A2.

The CCS values of compounds 1–5 were obtained by TIMS-TOF-MS in positive ionisation mode. The acquisition of CCS values in negative mode resulted in several deprotonated species and adducts with identical *m*/*z* values, which could be separated in IMS dimension (data not shown). Therefore, the positive mode was preferred. The CCS values for [M+H]^+^ were 227.6 Å^2^ (B1), 226.4 Å^2^ (B2), 224.3 Å^2^ (B3), and 216.4 Å^2^ (B4), while the A-types A1 (1) and PC A2 (2) have CCS values of 235.7 Å^2^ for 1 and 239.9 Å^2^ for 2 (Table 2). The A- and B-types can be differentiated based on their CCS values, which were higher for the A-types than for the B-types. Therefore, the CCS value allowed for rapid differentiation beyond the *m*/*z* ratio. Wang et al. (2020) also demonstrated that A-types had larger CCS values than B-types, which is assumed to be a result of the additional ether bridge. In the study of Sousa Dias et al. (2022), B1–B4 showed 1/*K*_*o*_ values between 1.067 and 1.091, which aligns with the values determined in this study (Table 2). The predicted CCS values from https://ccsbase.net (Ross et al., 2020) were compared with the experimentally measured CCS values and showed an overall opposite trend: The predicted CCS values were higher for the B-types and lower for the A-types. Due to the inverted pattern, the predicted CCS values provided insufficient differentiation of the linkage type (Table 2).

### 3.2 Oxidation products of B3 and B4

The oxidation of the C4*α*→C8 linked B3 and B4 dimers led to two main oxidation products. Oxidation product 1 (3) required less external energy with optimal conditions of 25.0 °C, 10 min, and a ratio of B3 to DPPH radical of 9:50 (*n*/*n*) (Table 1). In contrast, oxidation product 2 (4) required 314 min and 75.0 °C for optimal production with a ratio of B3 to DPPH radical of 9:12.5 (*n*/*n*).

The two main oxidation products of B3 (Figure S1 C, D) were successfully synthesised on a preparative scale and characterised by NMR spectroscopy (Table 4, Fig. S6–19). The oxidation product 1 (3) of B3 (Figure S1 C) represented approx. 38 % (determined chromatographically at λ = 280 nm) of all reaction products, and a total yield of 11.8 % was achieved. Not converted B3 was approx. 48 % after the reaction.

The second main oxidation product of B3 (4) was synthesised under optimal conditions (Table 1) by approx. 22 % (4; λ = 280 nm). In this reaction approach, the oxidation product 1 of B3 (3) was also formed with about 14 % (λ = 280 nm), and approx. 22 % of the unreacted B3 was detected after the reaction (Figure S1 D). Nevertheless, the target compounds could be purified through preparative HPLC with a yield of 3.43 % and the unconverted B3 was recovered.

Dimer B4 was obtained by semi-synthesis in lower quantities, as it contains (+)-catechin as the upper unit. Until now, no polymeric proanthocyanidin has been found in nature that consists only or mainly of (+)-catechin units (Esatbeyoglu et al., 2010b). Consequently, during the semi-synthesis with the addition of (−)-epicatechin as a nucleophile, the formation of B4 and B2 were observed. These two compounds exhibit similar chromatographic retention behaviours. The limited availability of natural products containing a high amount of (+)-catechin in the polymer, as well as the challenging isolation of B4 on a preparative scale due to the similar elution behaviour of B2 and B4, resulted in B4 being obtained with yields below 1 %. The oxidation and purification processes also caused losses, which is why only one of the two main oxidation products of B4 was isolated. According to Fischer et al. (2025), the formation rate of oxidation product 2 of B4 is approx. 25 % higher than that of oxidation product 1 of B4. The oxidation of B4 at 53.7 °C for 360 min with a DPPH radical to B4 ratio of 9/12.5 (*n*/*n*) resulted in the formation of compound 5, which was chromatographically detected at λ = 280 nm with approx. 32 % and unreacted B4 about 19 % after the reaction (Figure S1 E).

A comparison of the ^1^H and ^13^C NMR spectra of A1 (1) and A2 (2) with the oxidation products from B3 and B4 (3–5) (Table 4; Fig. S2–7, 14, 15, 22, 23) revealed that three carbons with δ_C_ at approx. 150 ppm, which are assigned to the carbons of the D-ring in the case of A-type linked PCs (C5t, C7t and C8at), were absent. A deshielding of these resonances, resulting in a chemical shift of about δ_C_ = 190 ppm, δ_C_ = 203 ppm, and δ_C_ = 170 ppm, respectively, was detected (Table 4). This suggests that the DPPH radicals led to the oxidation of B3 and B4 at the D-ring. The two resonances at δ_C_ = 190 ppm and δ_C_ = 203 ppm indicate that the hydroxyl groups at C7t–OH and C5t–OH were converted into carbonyl carbons.

The resonances from the carbonyl carbons were assigned using ^1^H–^13^C HMBC NMR experiments (Fig. 3 B). As a result of the carbonyl formation, the aromatic system of the D-ring was disturbed. This hypothesis was supported by the detection of a secondary carbon resonance identified by multiplicity-edited ^1^H–^13^C HSQC NMR spectrum and the absence of the singlet of H6t in the ^1^H NMR spectrum, which were detected for A1 (1) and A2 (2) (Table 3). Furthermore, since the H6t hydrogens only interact with each other and there were no other hydrogens in the vicinity, the position C6t consists of two hydrogens with a doublet. The assignment of the H6t resonance was further confirmed by ^1^H–^13^C HMBC correlations with the carbonyl carbons of H7t and H5t (Fig. 3 B–D). Moreover, the C8t is a quaternary carbon (spiro-carbon), in contrast to compounds 1 and 2. This was supported by the fact that H6t showed no coupling to H8t in the ^1^H NMR spectrum (δ_C_ = 68.8 ppm (3); δ_C_ = 68.6 ppm (4); δ_C_ = 68.3 ppm (5)).

There were also significant differences in the B-ring of the oxidation products 1 (3), 2 (4) of B3, and the oxidation product (5) of B4 compared to A1 (1) and A2 (2). The B-ring has only two proton signals as singlets. Moreover, two carbons in that B-ring had an δ_C_ shift typical for a hydroxyl group at approx. 145 ppm, suggesting an *o*-dihydroxylated aromatic ring system. Both signals corresponding to the protons on the B-ring form singlets which indicated that C6u′ was a quaternary carbon atom as determined by multiplicity-edited ^1^H–^13^C HSQC NMR spectrum. The fact that C6u′ and C8t were quaternary carbons indicated that a C–C single bond was formed between C6u′ and C8t during oxidation. This showed that the oxidation led to the formation of a spiro-linked product due to a suspected spirocyclisation (Fig. 1 B and C). This could be verified by the ^1^H–^13^C HMBC correlation of H4u to C8t and C6u' (Fig. 3 B–D). Hayashi et al. (2018) reported the formation of spiro-linked PCs due to the copper (II) chloride-induced oxidation of a C-(4*α*→8)-C dimer (B3). Moreover, Kataoka et al. (2024) also reported the formation of similar spiro-linked products, which occurs by the oxidation of B4 using CuCl_2_. Thereby, the *o*-dihydroxyl structure of the B-ring is oxidised to an *o*-quinone. Subsequently, an intramolecular 1,4-nucleophilic reaction occurs between the D-ring and the B-ring, resulting in the formation of a spiro ring system and the loss of the aromatic ring system of the D-ring. Furthermore, the opening of the F-ring led to the observation of epimeric compounds, which exhibited a change in the stereochemistry of C2t, as previously published by Hayashi et al. (2018).

Hayashi et al. (2018) and Kataoka et al. (2024) also described the formation of other spiro-linked oxidation products, which are formed after the generation of the additional C–C single bond by rearrangement of a new pyran ring involving the hydroxyl group of C5t (Hayashi et al., 2018; Kataoka et al., 2024). These products were not obtained as main oxidation products in our study.

The reaction changed the absolute configuration of the stereogenic centres of the formed spiro-linked PCs. The F-ring of the terminal unit of the product (3) exhibited a *trans*-configuration similar to the educt B3, with ^3^*J*_HH_ = 9.38 Hz between H2t and H3t (Fig. 3 B). However, ^1^H–^1^H ROESY correlations revealed that the observed H2t/H3t configuration was 2S, 3R, as for a (−)-catechin unit (Fig. S11). This differs from the educt B3, which has as terminal (+)-catechin unit and a configuration of 2R, 3S. This finding was attributed to the three-dimensional interaction between H5u′ and H2t with a distance of 3.89 Å as determined from the structure obtained after geometry optimisation using DFT calculations (B3LYP/6-31G(d)), including the solvent interaction of acetone using the CPCM (Fig. 3 B).

An increased reaction time and temperature resulted in an enhanced formation of product 2 of B3 (4). This product had a *cis*-configuration between H2t–H3t (^3^*J*_HH_ = 1.28), indicating that changing the reaction conditions caused a change in the configuration of the hydrogen atoms at the F-ring (Fig. 3 C). The ^1^H–^1^H ROESY correlation showed that the configuration of the H2t–H3t was a 2R, 3R-configuration, analogue to an (−)-epicatechin unit since the H6t′ has a three-dimensional interaction with the H5u' (4.16 Å) (Fig. S19). H5u′ also showed a correlation to the proton of the hydroxyl group at H3t position (2.94 Å). These distances were obtained from the structure after geometry optimisation as described above (Fig. 3 C).

The oxidation product of B4 (5) exhibited a *trans*-configuration of H2t and H3t as revealed by the coupling constants (^3^*J*_HH_ = 9.45 Hz, Table 4). ^1^H–^1^H ROESY correlation verified the *trans*-configuration of H2t and H3t with 2R, 3S (Fig. 3 D, Fig. S27) due to the correlation between H5u′ and H3t (3.62 Å, optimised by B3LYP/6-31G(d) method in acetone). The higher energy input during the reaction appeared to lead to isomerisation of the *cis*-configuration of H2t and H3t from the educt B4 to a *trans*-configuration. This was also the case for product 2 of B3 (4). Here, the B3 educt had a *trans*-configuration, and the formed product contained a *cis*-configuration (4). In the formation of product 1 of B3 (3), where a low energy input was required, the *trans*-configuration was not changed, only the absolute configuration of the stereogenic centres was changed.

A special feature of the isolated products (3–5) was that the protons in the upper unit of the C-ring showed a rigid W-configuration between H4u(quasi-eq)-C–CH3u(eq)–C-H2u(eq). Here, a ^4^*J*_HH_ long-range coupling was detected compared to the C-ring of the upper unit from A-type A2 (2) (no hydrogen at C2u position; section 3.1), and therefore generated a dd multiplicity (*J*_HH_ = 3.03, 1.92 Hz) of the H4u of compound 3 due to the interaction with H3u and H2u (Table 4). These H4u(quasi-eq)-C–CH3u(eq)–C-H2u(eq) configurations of the upper unit were identical for compounds 3, 4, and 5.

The absolute configuration of C4u from the educt B3 was a 4S-configuration, resulting in an α-orientation of the interflavan bond. To verify the absolute stereochemistry of compounds 3, 4, and 5, CD measurements were performed in addition to the ^1^H–^1^H ROESY NMR experiments. The CD spectra of the isolated oxidation products 3–5 (3, [θ]_211_ = +212,078 with a shoulder at [θ]_238_ = +50,043; 4, [θ]_210_ = +204,341 with a shoulder at [θ]_239_ = +27,489; 5, [θ]_212_ = +133,096 with a shoulder at [θ]_231_ = +40,760) showed a positive Cotton effect between λ = 200–220 nm (^1^L_a_ band), indicating a 4R-configuration (*β*-orientation) (Fig. 4 C–E) (Barrett et al., 1979; Slade et al., 2005). A negative Cotton effect for the ^1^L_B_ band at approx. λ = 280 nm was also detected for compounds 3 (294 nm), 4 (289 nm), and 5 (290 nm). However, the isolated oxidation products of B3 and B4 exhibited a positive Cotton effect between λ = 200–220 nm and were assumed to have a 4S-configuration. Due to the deviation and the lack of existing studies on the Cotton effect of such spiro-linked PCs, TD-DFT-based calculations of the CD spectrum of the isolated compounds 3–5 were performed to verify the correct steric assignment obtained from the NMR analysis. For this purpose, a geometry optimisation was first performed using the B3LYP functional and def2-SVP basis sets, incorporating the interactions of methanol using CPCM, since methanol was used as solvent for the CD measurements. For CD calculation the basis-set was changed to def2-TZVP. The predicted CD spectra of the tree compounds (3–5) showed the same trends of the Cotton effects but exhibited a slight hypochromic shift (Fig. 4 F–H). The prediction also resulted in a positive Cotton effect for the ^1^L_a_ band and a negative Cotton effect for the ^1^L_B_ band. In the case of the spiro-linked oxidation products, the application of CD spectroscopy led to a positive Cotton effect for ^1^L_a_ band for the spiro-linked products (3–5) with a 4S-configuration and not to a negative Cotton effect as suspected for the B-type PCs (Barrett et al., 1979). The 4S-configuration was also consistent with the detected NMR correlations (Fig. 3 B–D).

The comparison of the CCS values of compounds 1–5 and the used B-types were determined by TIMS-TOF-MS in positive ionisation mode and showed that the A-types (1, 2), B-types (B1–B4), and spiro-linked PCs (3–5) could be differentiated as separate groups based on the CCS values (Table 2). Since the spiro-linked compounds (3–5) showed CCS values of approx. 220 Å^2^ (220.2 Å^2^ (3), 219.8 Å^2^ (4), and 220.3 Å^2^ (5)), and the A-types showed CCS values of 235.7 Å^2^ (1) and 235.9 Å^2^ (2), differentiation between the epimers was only possible by using their retention times in combination. Overall, the A-types led to higher CCS values than the spiro-linked PCs (3–5). It was shown that the additional spirocyclic ring system of 3–5 resulted in lower CCS values in the TIMS, presumably due to the smaller cross-section. In contrast to the experimental CCS values, the predicted values (https://ccsbase.net, Ross et al., 2020) were higher for spiro-linked PCs with approx. 234.8 Å^2^ (measured approx. 220 Å^2^) than for A-type PCs. Controversy, the actual measured CCS values for the A-types were higher than those for the spiro-linked PCs.

### 3.3 Antioxidant activity

In this study, four photometric assays were conducted to assess the antioxidant activity according to different mechanisms. Two radical-based assays (DPPH and TEAC) and two reducing power-based assays (CUPRAC and FRAP) were applied.

It is important to note that the isomerisation of compounds 3 and 4 occurs in solution, as described by Hayashi et al. (2018). Therefore, only the antioxidant activity between the A-types and the isomeric spiro-linked products was distinguished, since their isomerisation cannot be prevented at room temperature during measurements.

**Fig. 5:**
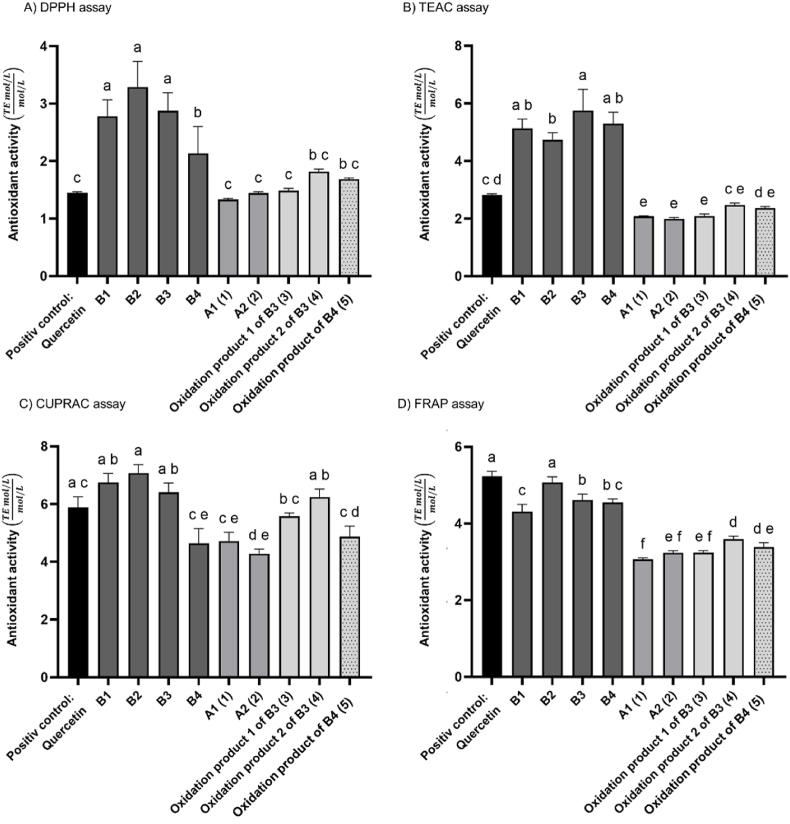
Radical scavenging properties were determined by A) DPPH assay and B) TEAC assay, as well as the reducing power by C) CUPRAC assay and D) FRAP assay of quercetin (positive control), B1–B4, A1 (1), A2 (2), oxidation product 1 of B3 (3), oxidation product 2 of B3 (4), and oxidation product of B4 (5). Results are given as a concentration-based ratio of the measured antioxidant activity as Trolox equivalents (mol/L) per mol/L substance; mean ± SD, n = 3; Shapiro-Wilk test followed by one-way ANOVA with Tukey's multiple comparison test. Compounds with different superscript letters show significant differences (*p <* 0.05).

B-type PCs showed the highest radical scavenging activity (Fig. 5 A and B), while the reducing power of the B-types and quercetin were overall comparable (Fig. 5 C and D).

In the DPPH and TEAC assays, the oxidation product 2 of B3 (4) and the oxidation product of B4 (5) showed the highest radical scavenging activity of all the oxidation products (1–5) (Fig. 5 A and B). The oxidation products (1–5) had a lower ABTS and DPPH radical scavenging activity than the B-type PCs B1–B4 and quercetin but higher than Trolox, since the ratio is higher than one. The antioxidant activity related to the reduction of a copper (II)-neocuproine complex in the CUPRAC assay was lower for A1 (1) and A2 (2) than for the spiro-linked compounds (3, 4), which showed a comparable activity to the B-types and quercetin (Fig. 5 C). These results demonstrated the high efficiency of spiro-linked PCs (3, 4) on the reduction of copper (II). The oxidation product of B4 (5), A1 (1), A2 (2), and B4 showed comparable activity in the CUPRAC assay. In the FRAP assay, a significantly lower reducibility of all compounds (1–5) compared to quercetin and the B-types were observed in reducing an iron (III)-TPTZ complex (Fig. 5 D). The observed discrepancy between the CUPRAC and FRAP assays for the spiro-products likely arises from the different chemical conditions and mechanisms underlying these antioxidant assays. The CUPRAC assay is performed at a pH of 7, whereas the FRAP assay is performed under acidic conditions at a pH of 3.6 (Apak et al., 2008). As the CUPRAC assay is performed under conditions that are almost physiological, it is preferred over the FRAP assay (Kukić-Marković, 2023).

The antioxidant effect of flavonoids, including the B-type PCs and their oxidation products (1–5), as well as quercetin, is attributable to the 3′,4′-dihydroxy position at the B-ring (PC, quercetin), the presence of a 3-hydroxyl group (PC) or 4-carbonyl group with the double bond between C2 and C3 (quercetin) (Rice-Evans et al., 1995, 1996; Zheng et al., 2017; Plumb et al., 1998). In this study, the radical scavenging activity of quercetin was lower than that of the B-types and generally comparable to that of the A-type PCs (Fig. 5 A and B). In the CUPRAC the activity was similar to that of the B-types and higher than that of the A-types (1, 2).

Various studies, including the present study, have shown that the A-types have a lower radical scavenging activity than the B-types (Plumb et al., 1998; Saint-Cricq De Gaulejac et al., 1999; Dong et al., 2013). The antioxidant activity of B1–B4 and A2 was determined by Plumb et al. (1998) using the TEAC assay. In this study, the B-type PCs exhibited higher ABTS radical scavenging activity than A2. Our results are consistent with Plumb et al. (1998). However, Dong et al. (2013) could not detect a significant difference in ABTS radical scavenging properties between A- and B-type PCs. In contrast, the DPPH assay revealed that the A-types had lower DPPH radical scavenging activity than the B-types, as observed in our study. The differences in the results may be explained by the different experimental procedures and varied evaluations.

The lower radical scavenging of the A-types is attributed to the absence of one hydroxyl groups compared to the B-types. Due to the ether bridge (C2u–*O*–C7t) present in the A-types, there is one less hydroxyl group on C7. Furthermore, the hydrogen at the C2 position, which is not present in the A-types due to the ether bridge, is linked to the ability to achieve DPPH radical abstraction (Dong et al., 2013). Depending on the assay, the spiro-linked products (3–5), especially compound 4, exhibited a higher radical scavenging (DPPH and TEAC assay) and reducing activity (CUPRAC assay) compared to compounds 1 and 2. In fact, the spiro-linked PCs (3–5) displayed a loss of the aromatic system on the D-ring attributable to the formed carbonyl carbons at positions 5t and 7t. This resulted in the loss of one hydroxyl group compared to the A-types (1, 2). The hydrogen at the C2 position seems to play a central role in explaining the differences in the antioxidant activity between the A-types and the oxidation products of B3 and B4 (3–5), rather than the number of hydroxyl groups in the D-ring. However, the existing literature also suggests that the H2u significantly influences radical scavenging alongside the o-dihydroxyl structure (Kondo et al., 2000; Dong et al., 2013). A DFT-based study of the antioxidant activity of catechin and epicatechin determined that the preferred mechanism in polar solvents is the SPLET mechanism, where an electron is transferred first, followed by the absorption of a hydrogen atom (Anitha et al., 2020). This highlights the importance of H2u in addition to the *o*-dihydroxyl structure. Further research using DFT to investigate the differences in antioxidant mechanisms among B-types, A-types, and spiro-linked procyanidins (PCs) would be valuable for achieving a deeper understanding of these mechanisms.

## 4 Conclusions

Preparative scale oxidation of PCs B1 and B2 using DPPH radical under optimised conditions (Fischer et al., 2025) yielded the corresponding A-type compounds A1 (1; yield 36.7 %) and A2 (2; yield 18.4 %), while B3 and B4 resulted in isomeric oxidation products with a spiro-carbon at C8t with yields of 11.8 % (3), 3.43 % (4), and 15.1 % (5), respectively. Low-temperature NMR measurements were crucial for improved resolution and accurate interpretation in comprehensive structural investigations. CCS values determined by TIMS-TOF provided a rapid and practical differentiation of B-type, A-type, and spiro-linked PCs by using their retention times in combination.

Compared to the A-types A1 (1) and A2 (2), the spiro-linked PCs (3–5) showed equal or even higher radical scavenging and reducing antioxidant activity. In terms of reducibility of a copper (II)-neocuproine complex, the oxidation products of B3 (3, 4) were comparable to the B-types and showed higher activity than the A-types. This study provides initial indications of the antioxidant effects of spiro-linked PCs, making spiro-linked oxidation products promising candidates for further bioactivity-related research.

## CRediT authorship contribution statement

**Annik Fischer:** Formal analysis, Investigation, Methodology, Validation, Visualization, Writing – original draft. **Helen Keller:** Investigation, Methodology, Validation, Visualization, Writing – review & editing. **Jörn Droste:** Methodology, Resources, Writing – review & editing. **Recep Gök:** Conceptualization, Investigation, Methodology, Resources, Supervision, Validation, Visualization, Writing – review & editing. **Tuba Esatbeyoglu:** Conceptualization, Funding acquisition, Methodology, Project administration, Resources, Supervision, Validation, Writing – review & editing.

## Declaration of competing interest

The authors declare that they have no known competing financial interests or personal relationships that could have appeared to influence the work reported in this paper.

## Data Availability

Data will be made available on request.
