# Supplementary material for: Structure elucidation and antioxidant activity of the radical-induced oxidation products obtained from procyanidins B1 to B4
Source: Curr Res Food Sci. 2025 Aug 9;11:101160. doi: 10.1016/j.crfs.2025.101160 (PMC12409800; doi:10.1016/j.crfs.2025.101160)
Supplement: Multimedia component 1 [file mmc1.docx]

**Structure elucidation and antioxidant activity of the radical‑induced oxidation products obtained from procyanidins B1 to B4**

Annik Fischer ^a^, Helen Keller ^a^, Jörn Droste ^b^, Recep Gök ^c^, and Tuba Esatbeyoglu ^a*^

^a^Department of Molecular Food Chemistry and Food Development, Institute of Food and One Health, Leibniz University Hannover, Am Kleinen Felde 30, 30167 Hannover, Germany

^b^ Institute of Organic Chemistry, Leibniz University Hannover, Schneiderberg 1B, 30167 Hannover, Germany

^c^Institute of Food Chemistry, Technische Universität Braunschweig, Schleinitzstraße 20, 38106 Braunschweig, Germany

***Corresponding author:**

Prof. Dr. Tuba Esatbeyoglu

Gottfried Wilhelm Leibniz University Hannover

Institute of Food and One Health

Department of Molecular Food Chemistry and Food Development

Am Kleinen Felde 30

30167 Hannover, Germany

Tel: +49 511 762 5589, Fax: +49 511 762 4927

E-mail: esatbeyoglu@foh.uni-hannover.de

**E-mails:**

T. Esatbeyoglu = esatbeyoglu@foh.uni-hannover.de

A. Fischer = fischer@foh.uni-hannover.de

H. Keller = keller@foh.uni-hannover.de

J. Droste = joern.droste@oci.uni-hannover.de

R. Gök = r.goek@tu-braunschweig.de

**ORCID:**

T. Esatbeyoglu: 0000-0003-2413-6925

A. Fischer: 0000-0002-1977-3949

H. Keller: 0009-0005-4661-0423

J. Droste: 0000-0003-4863-6115

R. Gök: 0000-0002-1231-8679

# List of figures:

[Figure S 1. HPLC‑PDA chromatograms at λ = 280 nm with the *m*/*z* values in negative mode after oxidation with DPPH radicals on a preparative scale: A) Oxidation of B1 at 61.4 °C for 77.2 min and a ratio of B‑type to DPPH radical of 9:28.8 (*n*/*n*). B) Oxidation of B2 at 66.9 °C for 10.0 min and ratio of B‑type to DPPH radical of 9:26.5 (*n*/*n*). C) Oxidation of B3: Optimisation of the peak at 5.15 min (*m*/*z* 575 [M−H]^−^) at 25.0 °C for 10.0 min and a ratio of B‑type to DPPH radical of 9:50 (*n*/*n*). D) Oxidation of B3: Optimisation of the peak at 3.75 min (*m*/*z* 575 [M−H]^−^) at 75.0 °C for 314 min and a ratio of B‑type to DPPH radical of 9:12.5 (*n*/*n*). E) Oxidation of B4: Optimisation of the peak at 4.57 min (*m*/*z* 575 [M−H]^−^) at 53.7 °C for 360 min and a ratio of B‑type to DPPH radical of 9:12.5 (*n*/*n*). 4](#_Toc204258871)

[Figure S 2. ^1^H NMR spectrum of procyanidin A1 (1) recorded at 14.1 T and 253.0 K in acetone‑*d_6_.* 5](#_Toc204258872)

[Figure S 3. ^13^C NMR spectrum of procyanidin A1 (1) recorded at 14.1 T and 253.0 K in acetone‑*d_6_.* 5](#_Toc204258873)

[Figure S 4. ^1^H NMR spectrum of procyanidin A2 (2) recorded at 14.1 T and 253.0 K in acetone‑*d_6_.* 6](#_Toc204258874)

[Figure S 5. ^13^C NMR spectrum of procyanidin A2 (2) recorded at 14.1 T and 253.0 K in acetone‑*d_6_.* 6](#_Toc204258875)

[Figure S 6. ^1^H NMR spectrum of the oxidation product 1 of B3 (3) recorded at 14.1 T and 253.0 K in acetone‑*d_6_.* 7](#_Toc204258876)

[Figure S 7. ^13^C NMR spectrum of the oxidation product 1 of B3 (3) recorded at 14.1 T and 253.0 K in acetone‑*d_6_.* 7](#_Toc204258877)

[Figure S 8. ^1^H–^1^H COSY spectrum of the oxidation product 1 of B3 (3) recorded at 14.1 T and 253.0 K in acetone‑*d_6_.* 8](#_Toc204258878)

[Figure S 9. ^1^H–^1^H TOSY spectrum of the oxidation product 1 of B3 (3) recorded at 14.1 T and 253.0 K in acetone‑*d_6_.* 8](#_Toc204258879)

[Figure S 10. ^1^H–^1^H ROESY spectrum of the oxidation product 1 of B3 (3) recorded at 14.1 T and 253.0 K in acetone‑*d_6_.* 9](#_Toc204258880)

[Figure S 11. Partial ^1^H–^1^H ROESY spectrum of the oxidation product 1 of B3 (3) recorded at 14.1 T and 253.0 K in acetone‑*d_6_*, showing the observed ^1^H–^1^H ROESY correlations relevant for the determining of sterochemistry. 9](#_Toc204258881)

[Figure S 12. ^1^H–^13^C HSQC spectrum of the oxidation product 1 of B3 (3) recorded at 14.1 T and 253.0 K in acetone‑*d_6_.* 10](#_Toc204258882)

[Figure S 13. ^1^H–^13^C HMBC spectrum of the oxidation product 1 of B3 (3) recorded at 14.1 T and 253.0 K in acetone‑*d_6_.* 10](#_Toc204258883)

[Figure S 14. ^1^H NMR spectrum of the oxidation product 2 of B3 (4) recorded at 14.1 T and 253.0 K in acetone‑*d_6_.* 11](#_Toc204258884)

[Figure S 15. ^13^C NMR spectrum of the oxidation product 2 of B3 (4) recorded at 14.1 T and 253.0 K in acetone‑*d_6_.* 11](#_Toc204258885)

[Figure S 16. ^1^H–^1^H COSY spectrum of the oxidation product 2 of B3 (4) recorded at 14.1 T and 253.0 K in acetone‑*d_6_.* 12](#_Toc204258886)

[Figure S 17. ^1^H–^1^H TOSY spectrum of the oxidation product 2 of B3 (4) recorded at 14.1 T and 253.0 K in acetone‑*d_6_.* 12](#_Toc204258887)

[Figure S 18. ^1^H–^1^H ROESY spectrum of the oxidation product 2 of B3 (4) recorded at 14.1 T and 253.0 K in acetone‑*d_6_* (diagonal suppression applied)*.* 13](#_Toc204258888)

[Figure S 19. Partial ^1^H–^1^H ROESY spectrum of the oxidation product 2 of B3 (4) recorded at 14.1 T and 253.0 K in acetone‑*d_6_*, showing the observed ^1^H–^1^H ROESY correlations relevant for the determining of sterochemistry. 13](#_Toc204258889)

[Figure S 20. ^1^H–^13^C HSQC spectrum of the oxidation product 2 of B3 (4) recorded at 14.1 T and 253.0 K in acetone‑*d_6_.* 14](#_Toc204258890)

[Figure S 21. ^1^H–^13^C HMBC spectrum of the oxidation product 2 of B3 (4) recorded at 14.1 T and 253.0 K in acetone‑*d_6_.* 14](#_Toc204258891)

[Figure S 22. ^1^H NMR spectrum of the oxidation product of B4 (5) recorded at 14.1 T and 253.0 K in acetone‑*d_6_.* 15](#_Toc204258892)

[Figure S 23. ^13^C NMR spectrum of the oxidation product of B4 (5) recorded at 14.1 T and 253.0 K in acetone‑*d_6_.* 15](#_Toc204258893)

[Figure S 24. ^1^H–^1^H COSY spectrum of the oxidation product of B4 (5) recorded at 14.1 T and 253.0 K in acetone‑*d_6_.* 16](#_Toc204258894)

[Figure S 25. ^1^H–^1^H TOSY spectrum of the oxidation product of B4 (5) recorded at 14.1 T and 253.0 K in acetone‑*d_6_.* 16](#_Toc204258895)

[Figure S 26. ^1^H–^1^H ROESY spectrum of the oxidation product of B4 (5) recorded at 14.1 T and 253.0 K in acetone‑*d_6_.* 17](#_Toc204258896)

[Figure S 27. Partial ^1^H–^1^H ROESY spectrum of the oxidation product 1 of B4 (5) recorded at 14.1 T and 253.0 K in acetone‑*d_6_*, showing the observed ^1^H–^1^H ROESY correlations relevant for the determining of sterochemistry. 17](#_Toc204258897)

[Figure S 28. ^1^H–^13^C HSQC spectrum of the oxidation product of B4 (5) recorded at 14.1 T and 253.0 K in acetone‑*d_6_.* 18](#_Toc204258898)

[Figure S 29. ^1^H–^13^C HMBC spectrum of the oxidation product 2 of B4 (5) recorded at 14.1 T and 253.0 K in acetone‑*d_6_.* 18](#_Toc204258899)

# Figures:


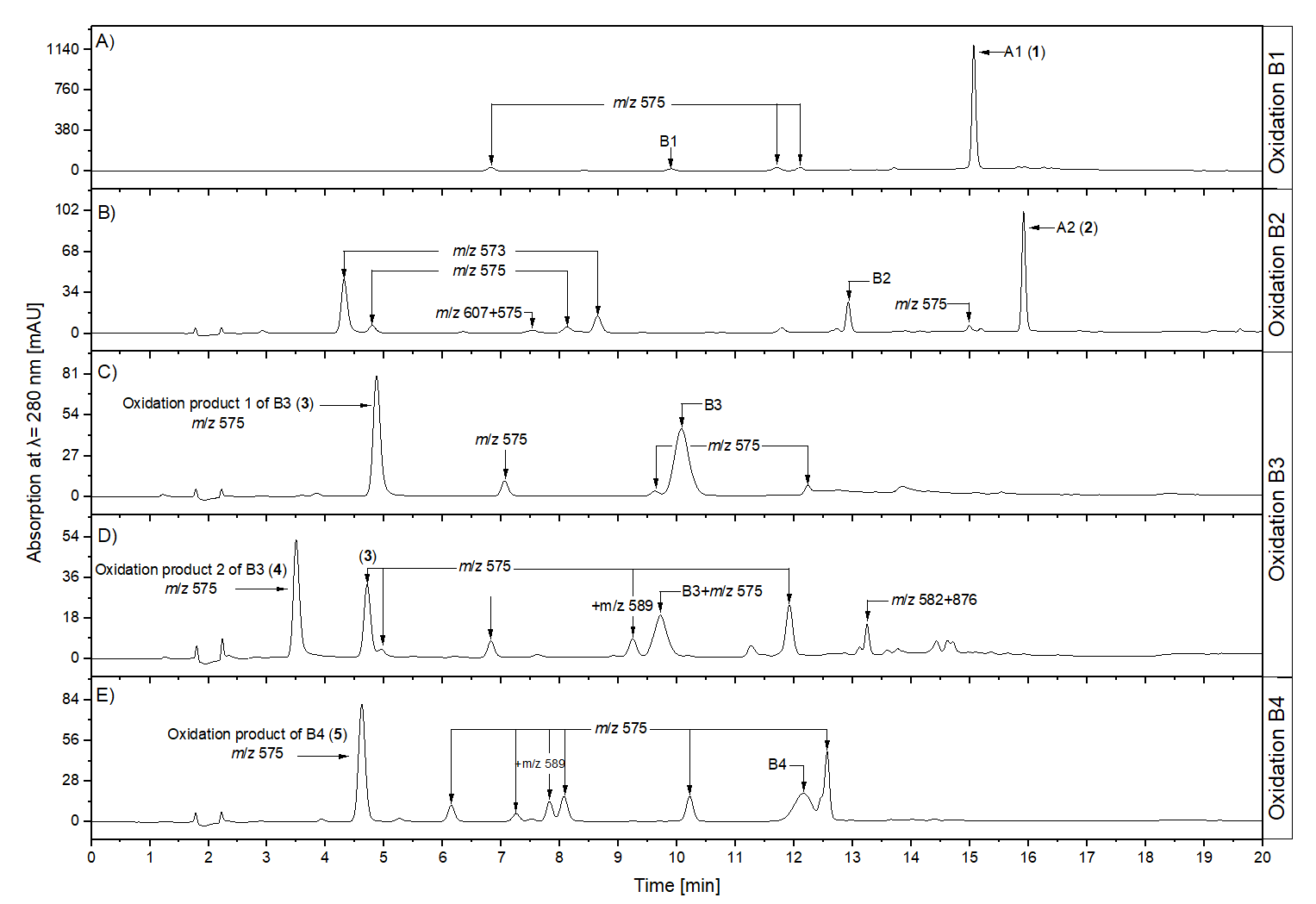


Figure S 1. HPLC‑PDA chromatograms at λ = 280 nm with the *m*/*z* values in negative mode after oxidation with DPPH radicals on a preparative scale: A) Oxidation of B1 at 61.4 °C for 77.2 min and a ratio of B‑type to DPPH radical of 9:28.8 (*n*/*n*). B) Oxidation of B2 at 66.9 °C for 10.0 min and ratio of B‑type to DPPH radical of 9:26.5 (*n*/*n*).
C) Oxidation of B3: Optimisation of the peak at 5.15 min (*m*/*z* 575 [M−H]^−^) at 25.0 °C for 10.0 min and a ratio of B‑type to DPPH radical of 9:50 (*n*/*n*). D) Oxidation of B3: Optimisation of the peak at 3.75 min (*m*/*z* 575 [M−H]^−^) at 75.0 °C for 314 min and a ratio of B‑type to DPPH radical of 9:12.5 (*n*/*n*). E) Oxidation of B4: Optimisation of the peak at 4.57 min (*m*/*z* 575 [M−H]^−^) at 53.7 °C for 360 min and a ratio of B‑type to DPPH radical of 9:12.5 (*n*/*n*).

All NMR spectra (Figure S 2–29) were processed with manual phase correction, multipoint baseline correction, and zero filling to 4k points (only ROESY).


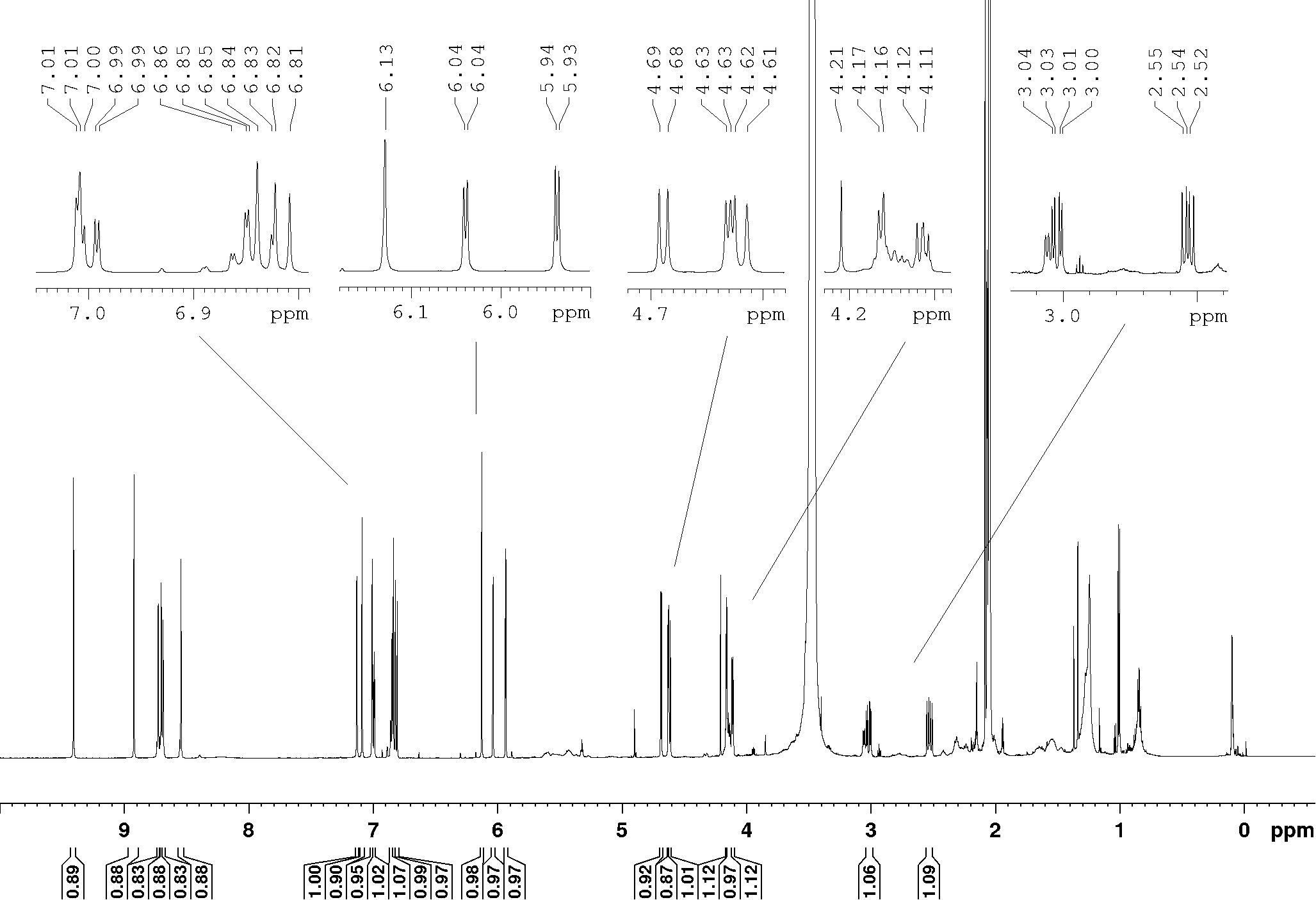


Figure S 2. ^1^H NMR spectrum of procyanidin A1 (1) recorded at 14.1 T and 253.0 K in acetone‑*d_6_.*


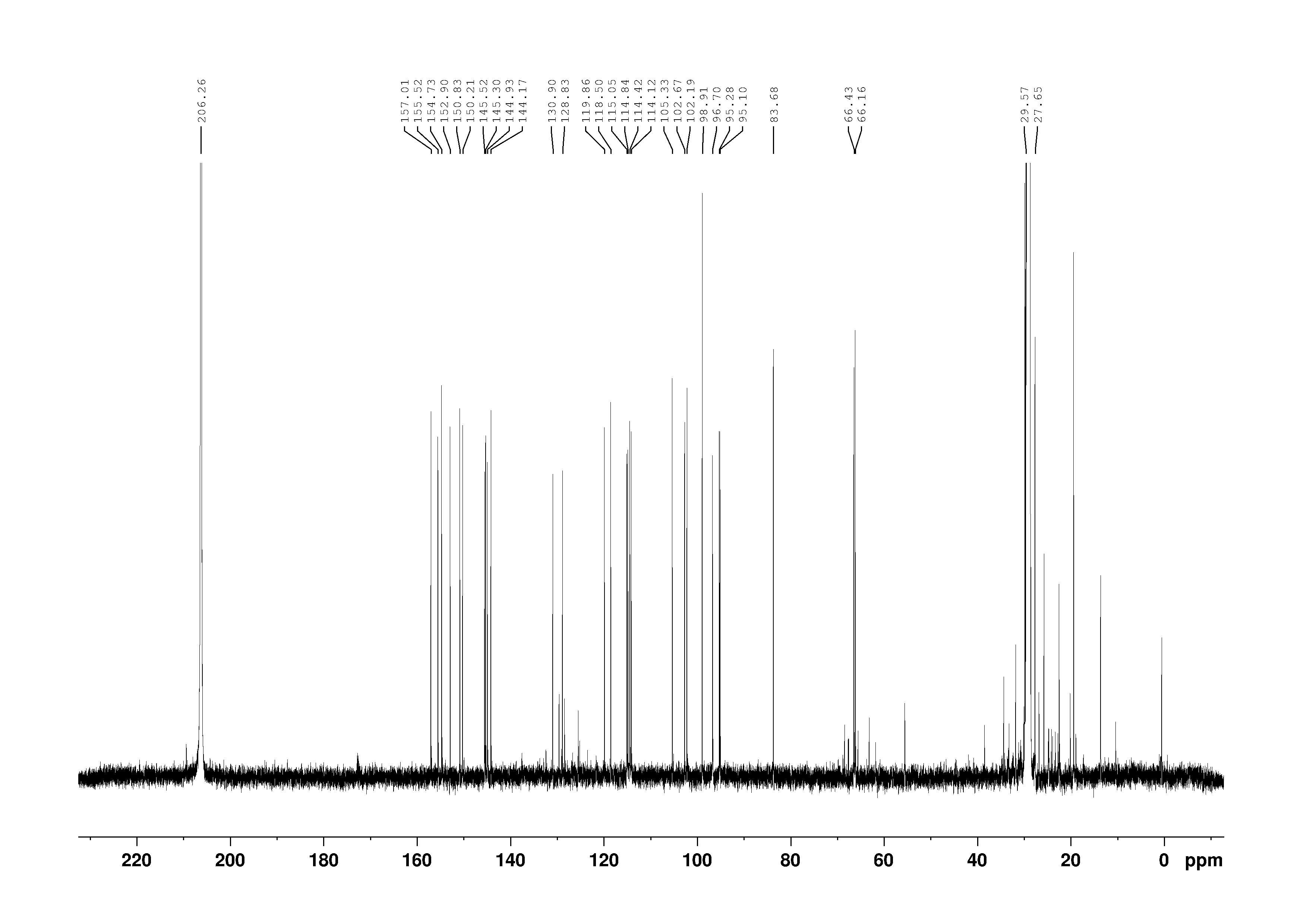


Figure S 3. ^13^C NMR spectrum of procyanidin A1 (1) recorded at 14.1 T and 253.0 K in acetone‑*d_6_.*


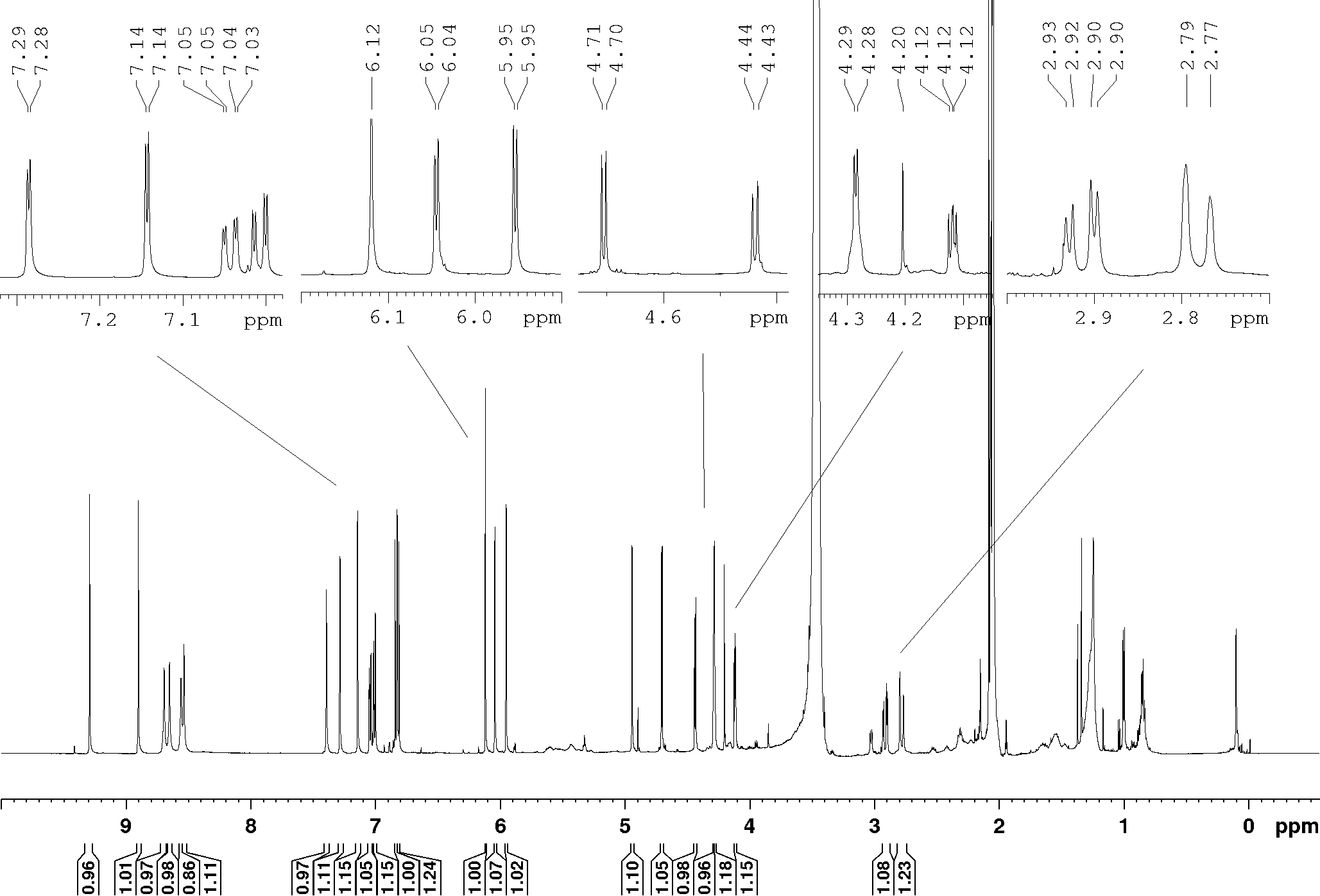


Figure S 4. ^1^H NMR spectrum of procyanidin A2 (2) recorded at 14.1 T and 253.0 K in acetone‑*d_6_.*


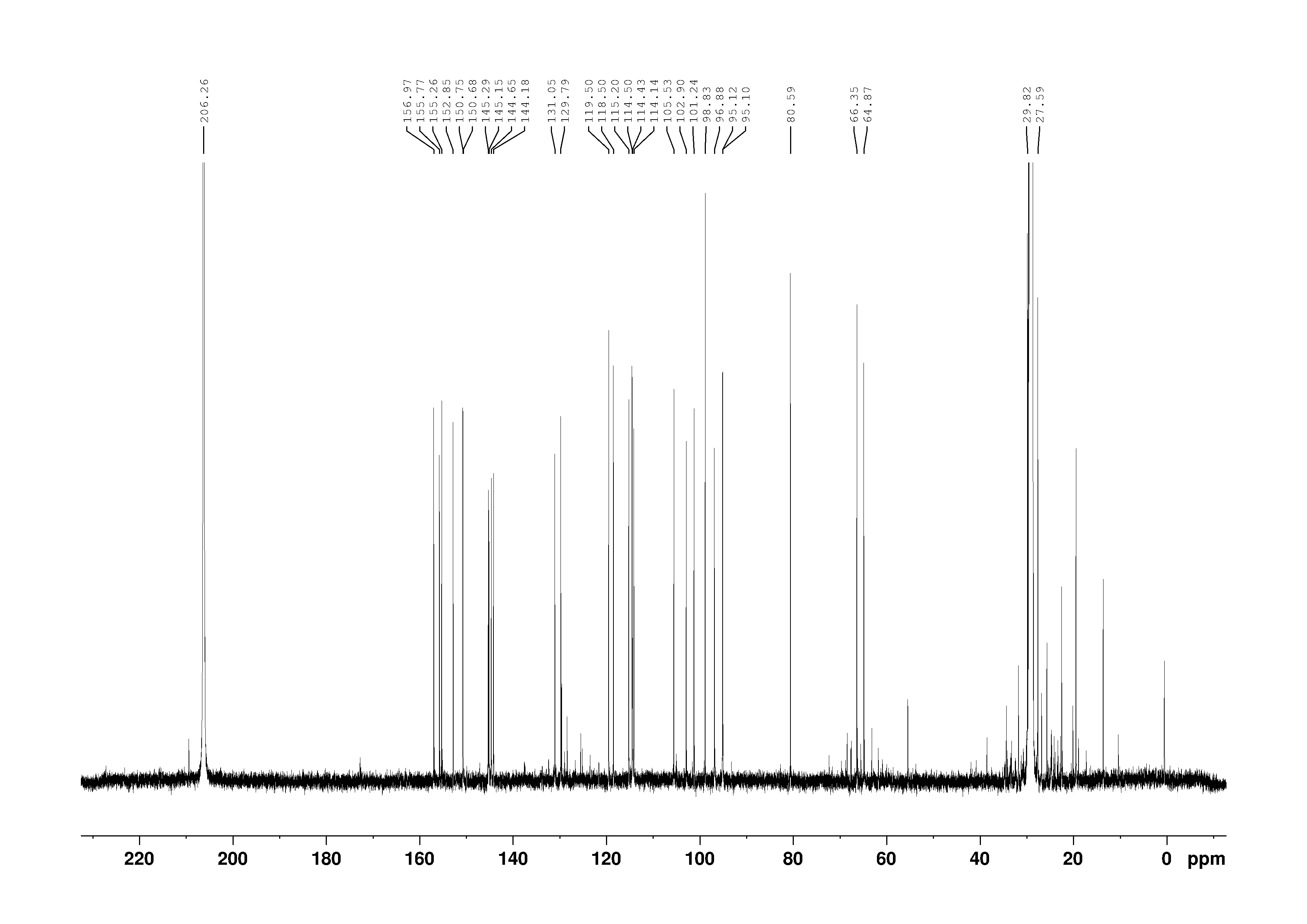


Figure S 5. ^13^C NMR spectrum of procyanidin A2 (2) recorded at 14.1 T and 253.0 K in acetone‑*d_6_.*


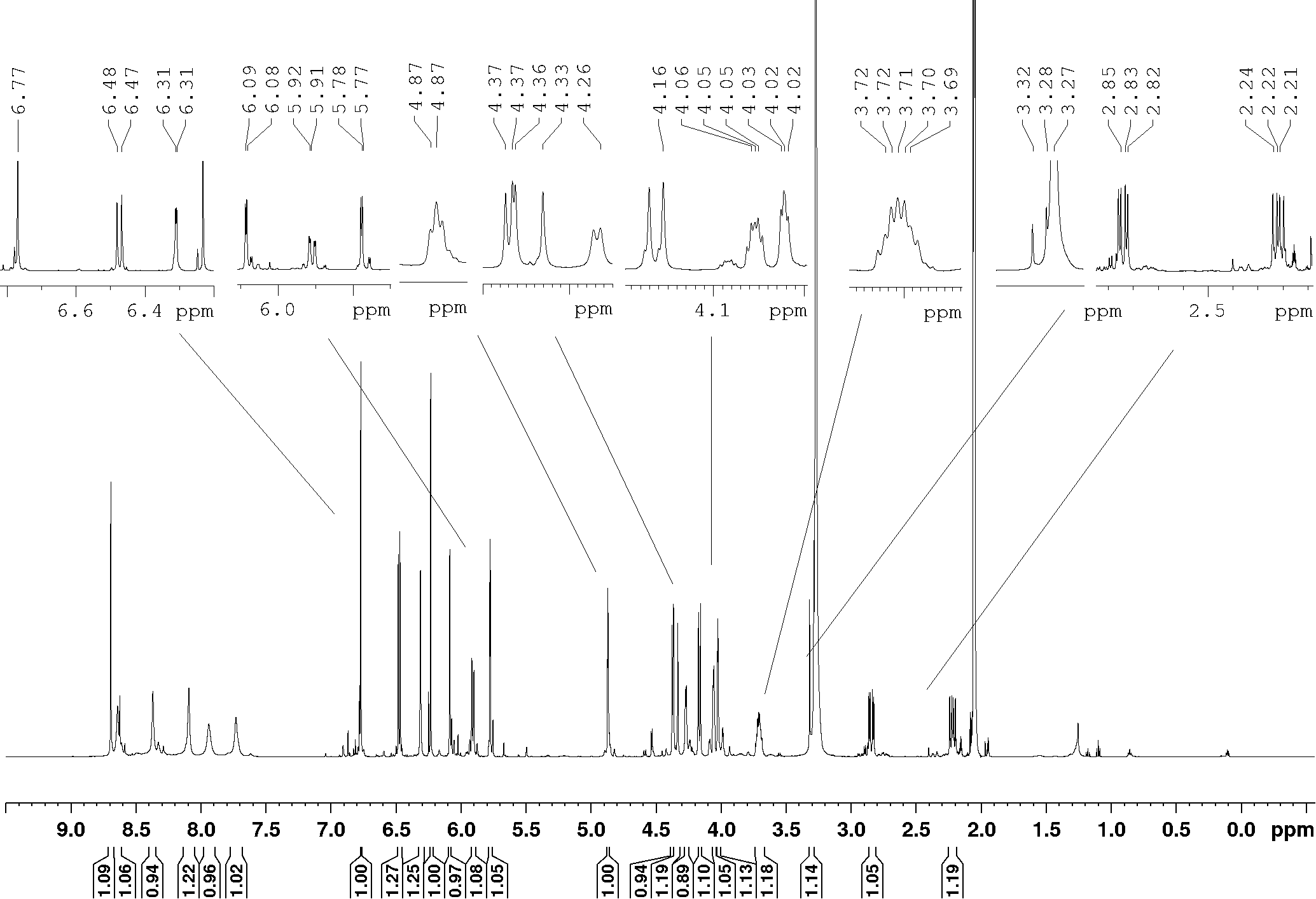


Figure S 6. ^1^H NMR spectrum of the oxidation product 1 of B3 (3) recorded at 14.1 T and 253.0 K in acetone‑*d_6_.*


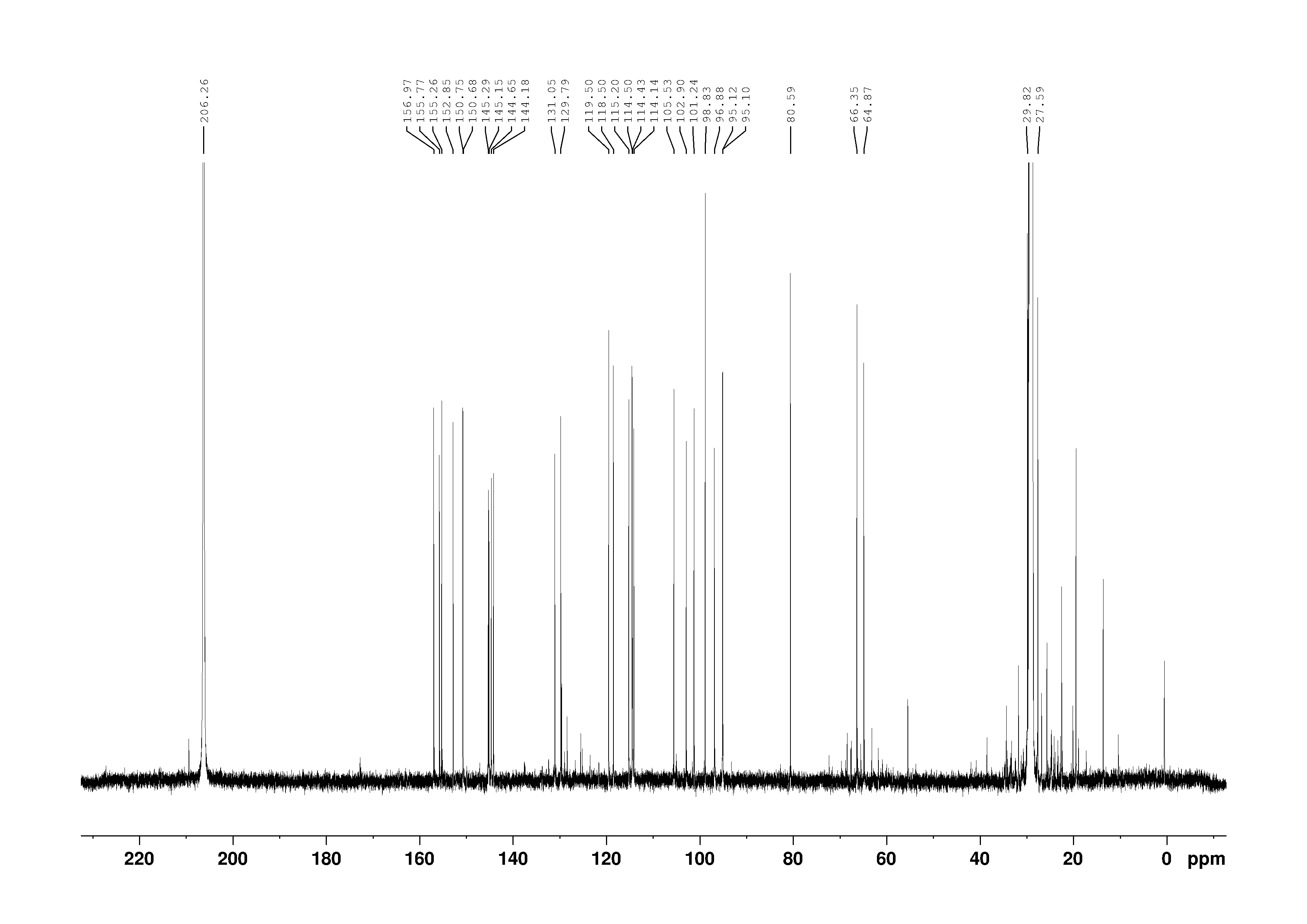


Figure S 7. ^13^C NMR spectrum of the oxidation product 1 of B3 (3) recorded at 14.1 T and 253.0 K in acetone‑*d_6_.*


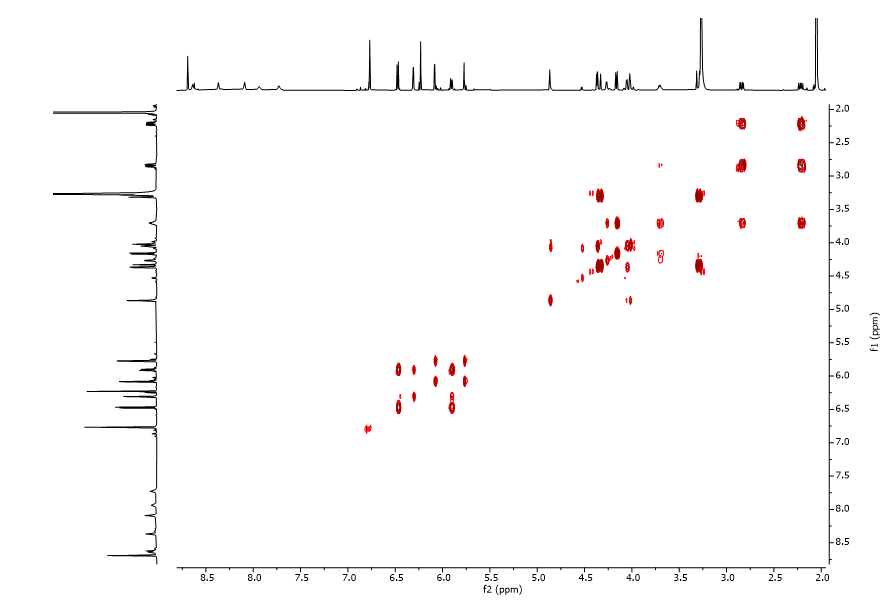


Figure S 8. ^1^H–^1^H COSY spectrum of the oxidation product 1 of B3 (3) recorded at 14.1 T and 253.0 K in acetone‑*d_6_.*


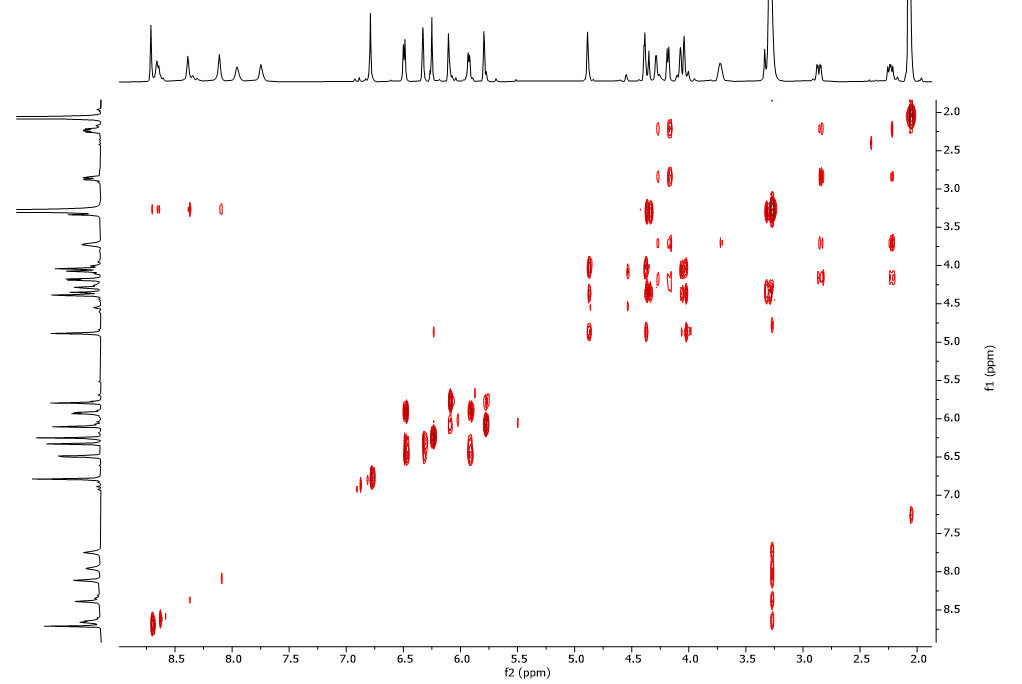


Figure S 9. ^1^H–^1^H TOSY spectrum of the oxidation product 1 of B3 (3) recorded at 14.1 T and 253.0 K in acetone‑*d_6_.*

Figure S 10. ^1^H–^1^H ROESY spectrum of the oxidation product 1 of B3 (3) recorded at 14.1 T and 253.0 K in acetone‑*d_6_.*


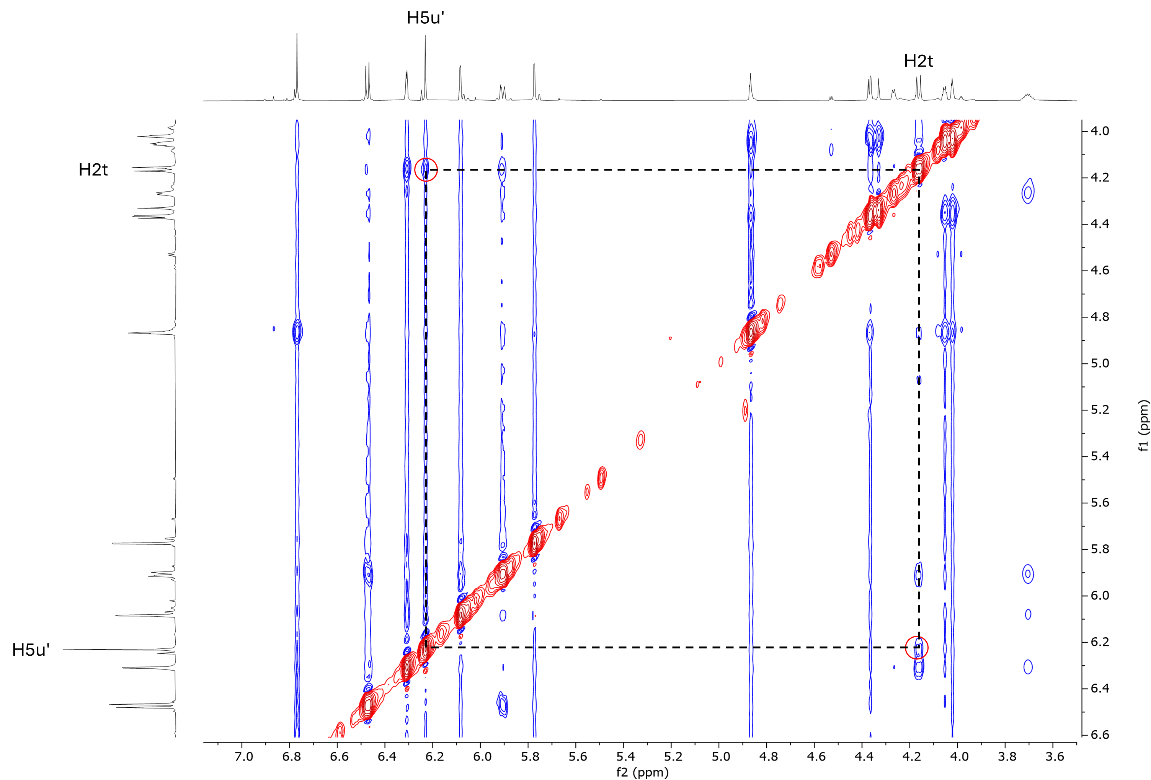


Figure S 11. Partial ^1^H–^1^H ROESY spectrum of the oxidation product 1 of B3 (3) recorded at 14.1 T and 253.0 K in acetone‑*d_6_*, showing the observed ^1^H–^1^H ROESY correlations relevant for the determining of sterochemistry.


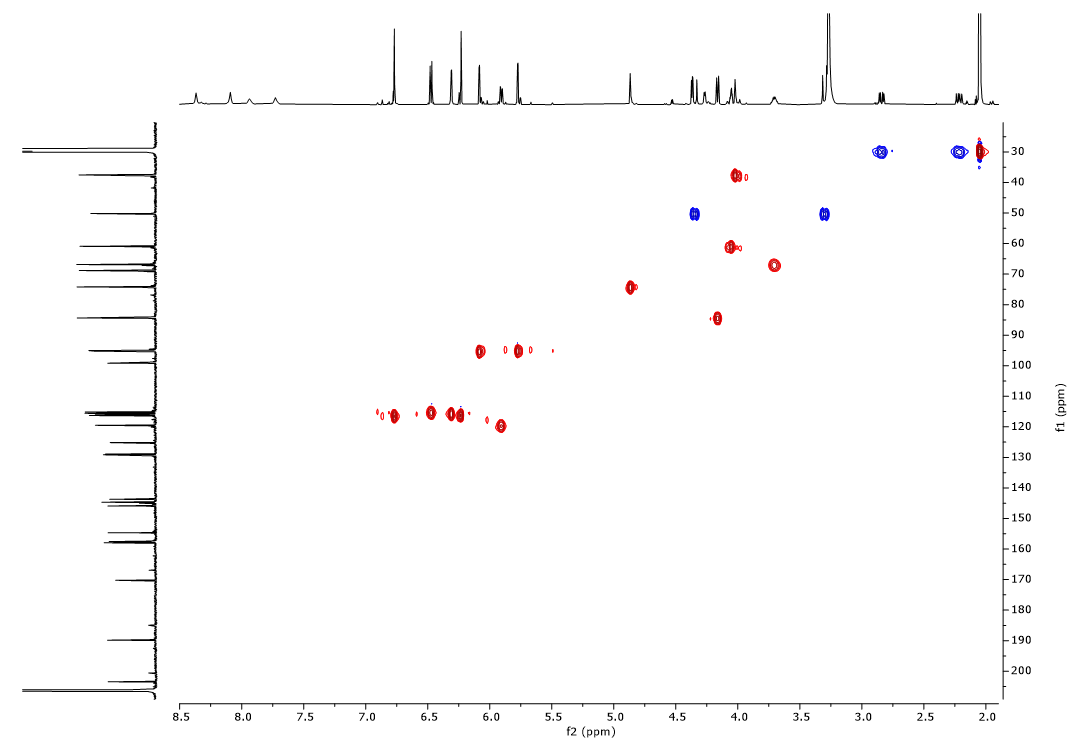


Figure S 12. ^1^H–^13^C HSQC spectrum of the oxidation product 1 of B3 (3) recorded at 14.1 T and 253.0 K in acetone‑*d_6_.*


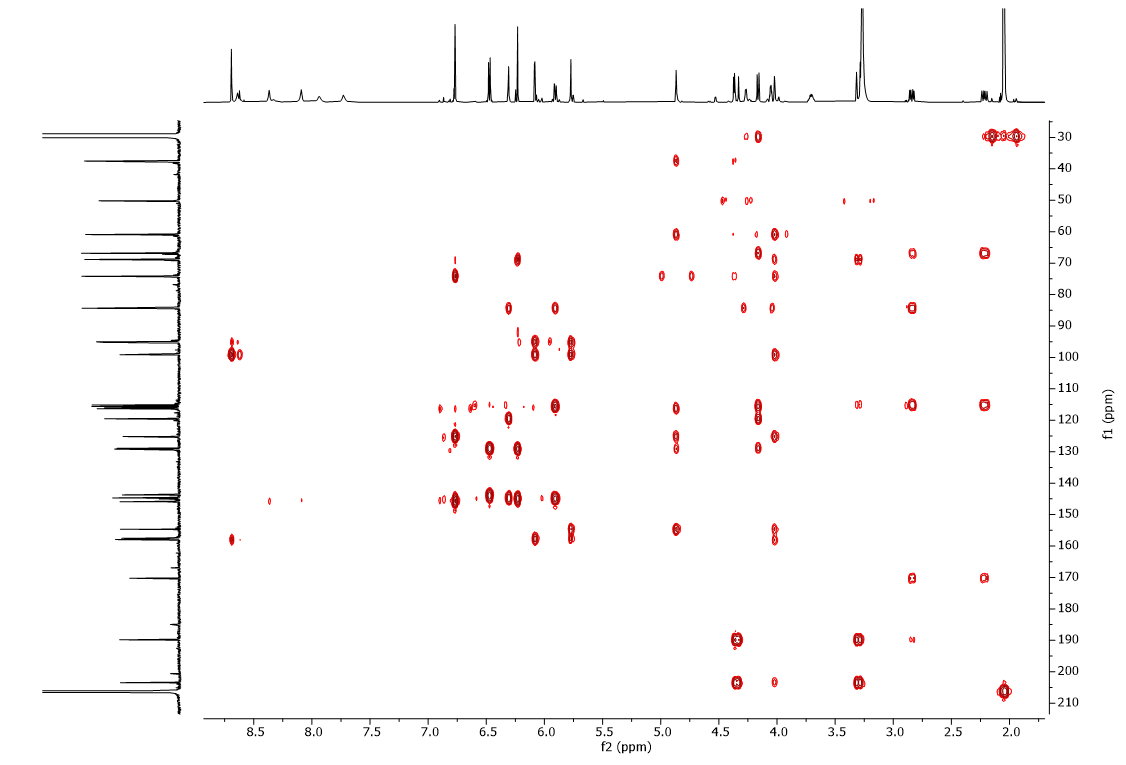


Figure S 13. ^1^H–^13^C HMBC spectrum of the oxidation product 1 of B3 (3) recorded at 14.1 T and 253.0 K in acetone‑*d_6_.*


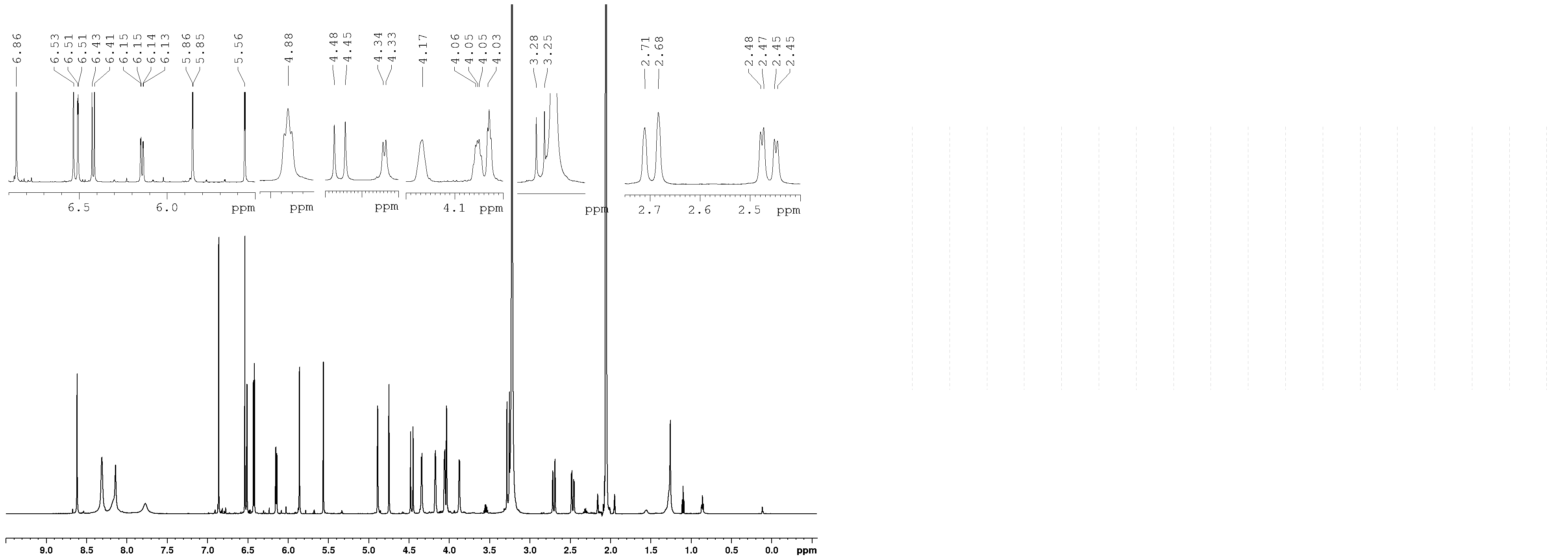


Figure S 14. ^1^H NMR spectrum of the oxidation product 2 of B3 (4) recorded at 14.1 T and 253.0 K in acetone‑*d_6_.*


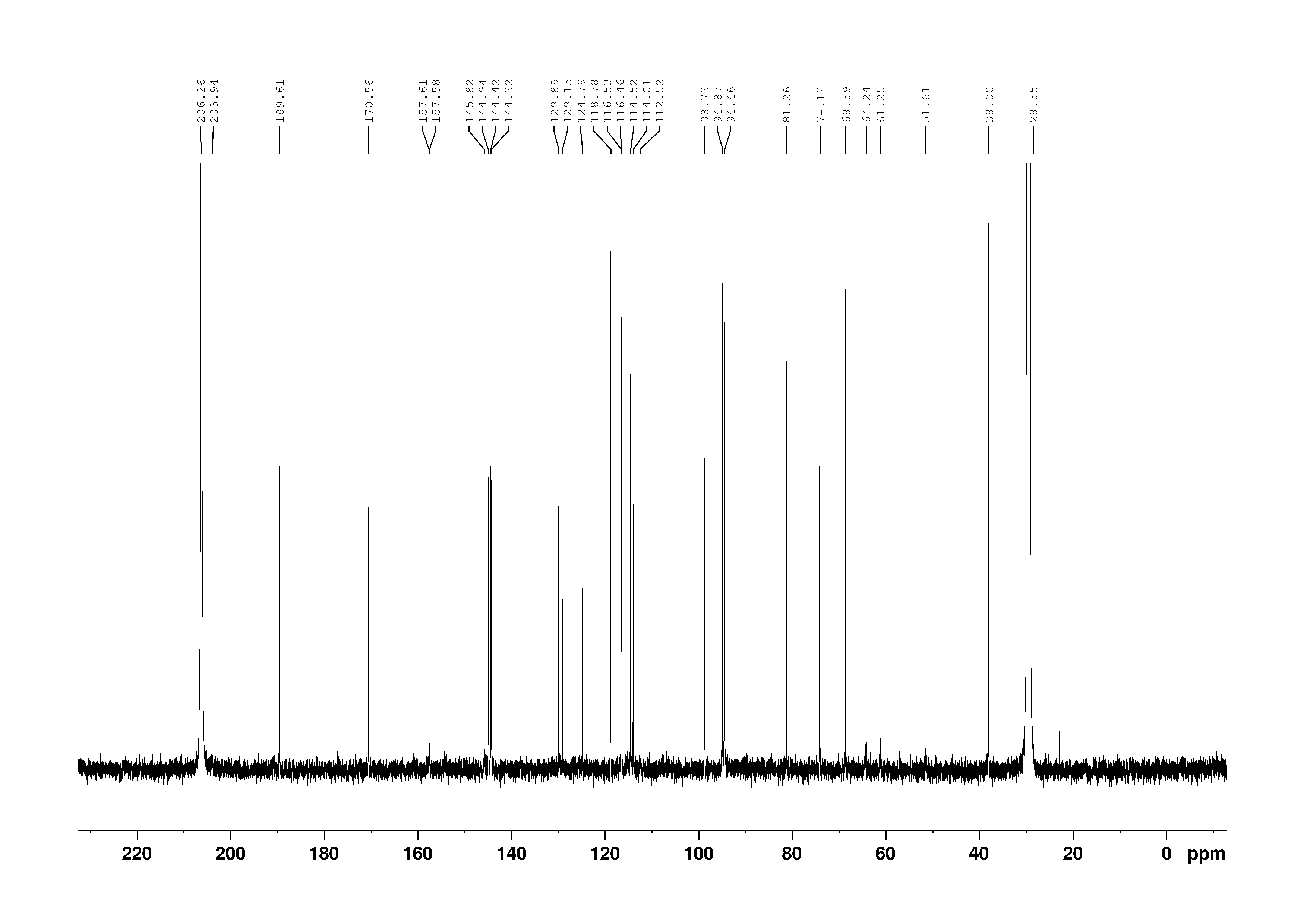


Figure S 15. ^13^C NMR spectrum of the oxidation product 2 of B3 (4) recorded at 14.1 T and 253.0 K in acetone‑*d_6_.*


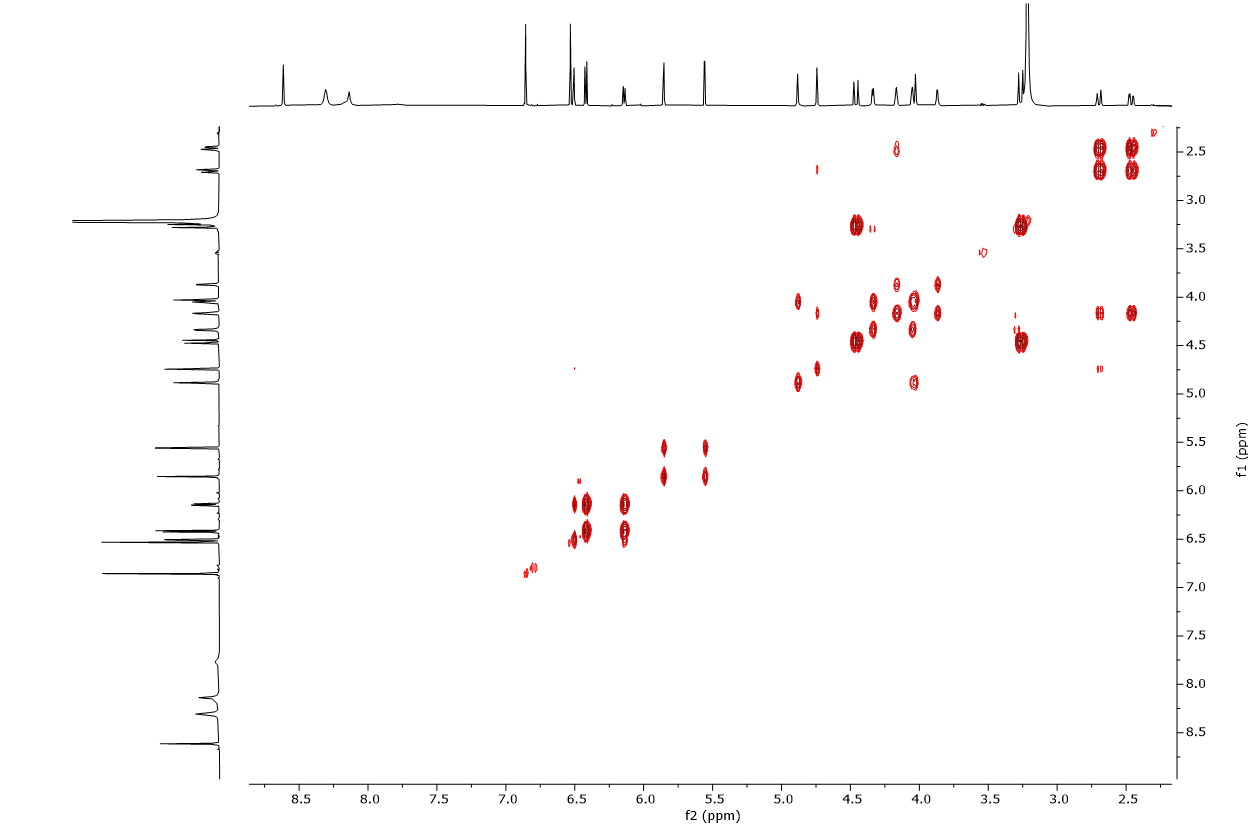


Figure S 16. ^1^H–^1^H COSY spectrum of the oxidation product 2 of B3 (4) recorded at 14.1 T and 253.0 K in acetone‑*d_6_.*


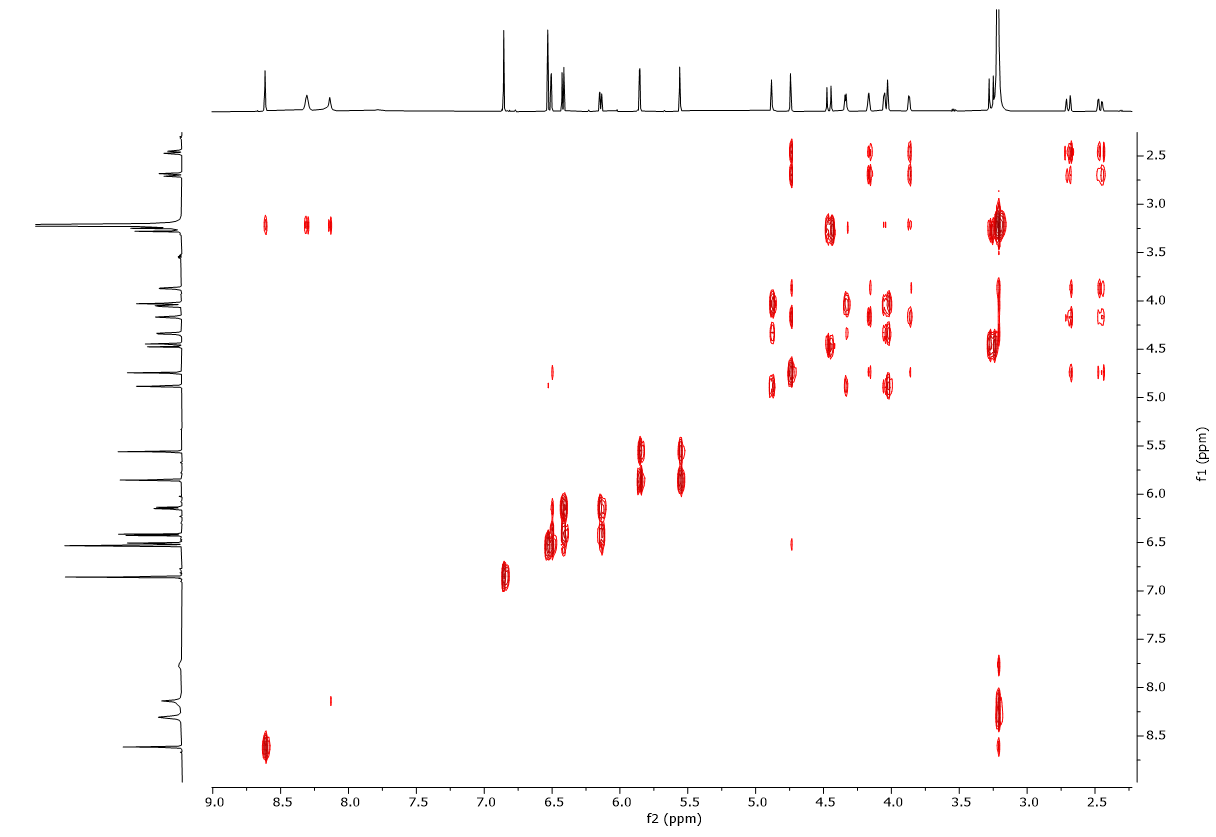


Figure S 17. ^1^H–^1^H TOSY spectrum of the oxidation product 2 of B3 (4) recorded at 14.1 T and 253.0 K in acetone‑*d_6_.*


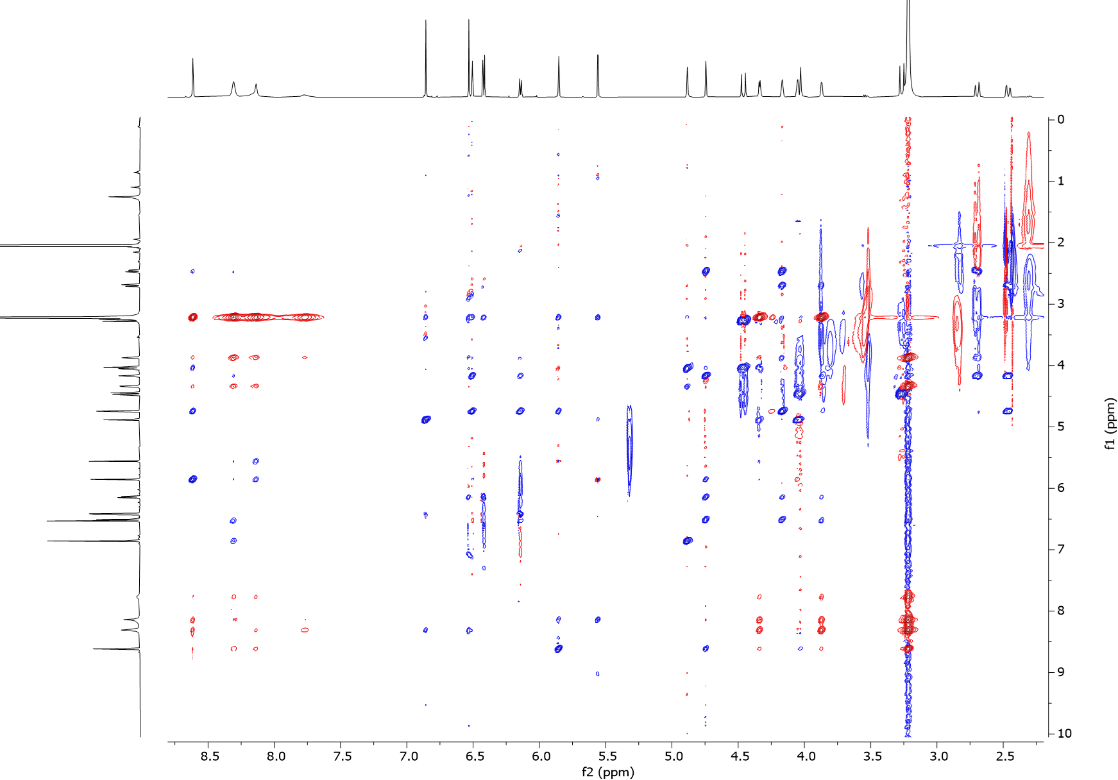


Figure S 18. ^1^H–^1^H ROESY spectrum of the oxidation product 2 of B3 (4) recorded at 14.1 T and 253.0 K in acetone‑*d_6_* (diagonal suppression applied)*.*


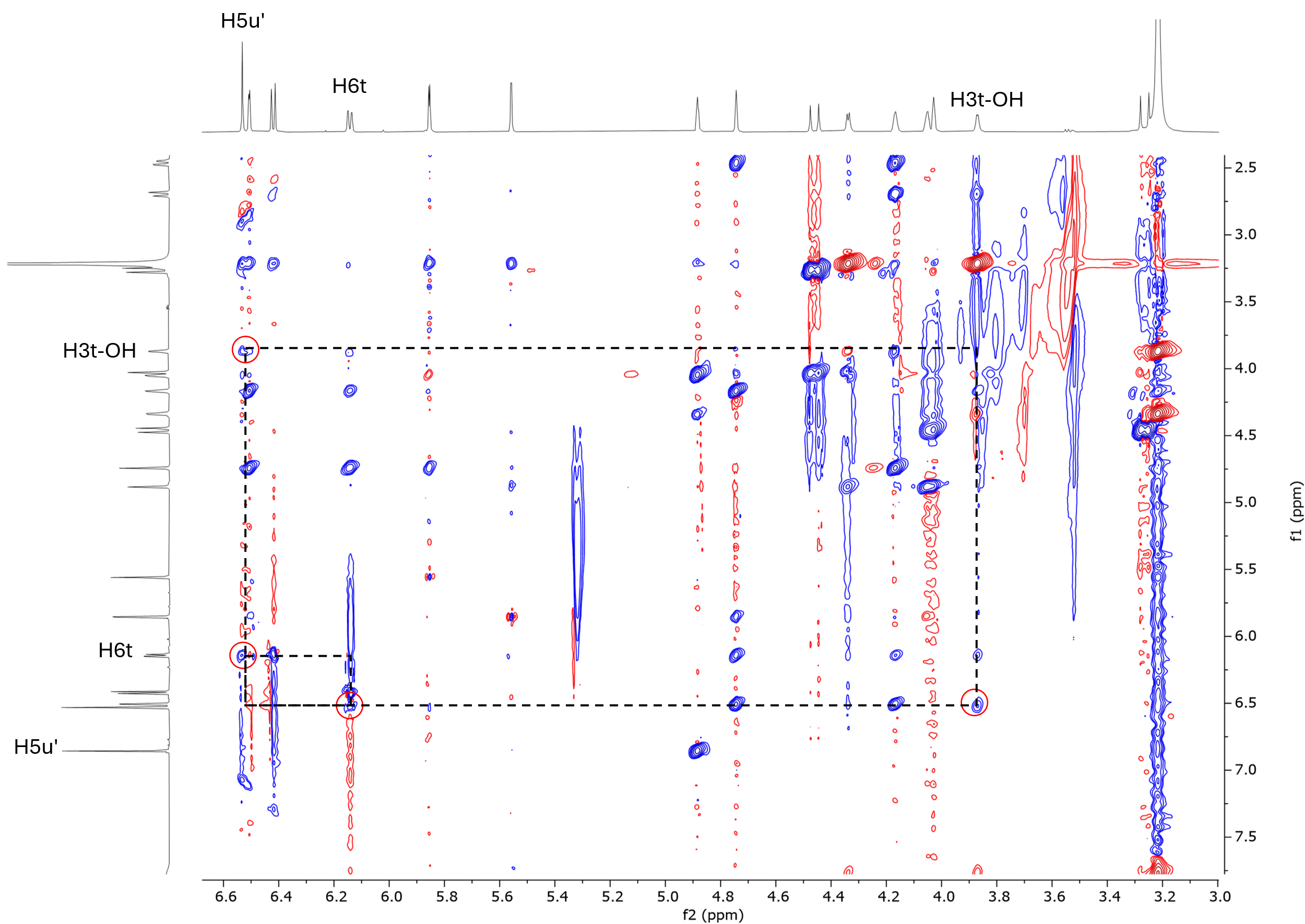


Figure S 19. Partial ^1^H–^1^H ROESY spectrum of the oxidation product 2 of B3 (4) recorded at 14.1 T and 253.0 K in acetone‑*d_6_* (diagonal suppression applied), showing the observed ^1^H–^1^H ROESY correlations relevant for the determining of sterochemistry.


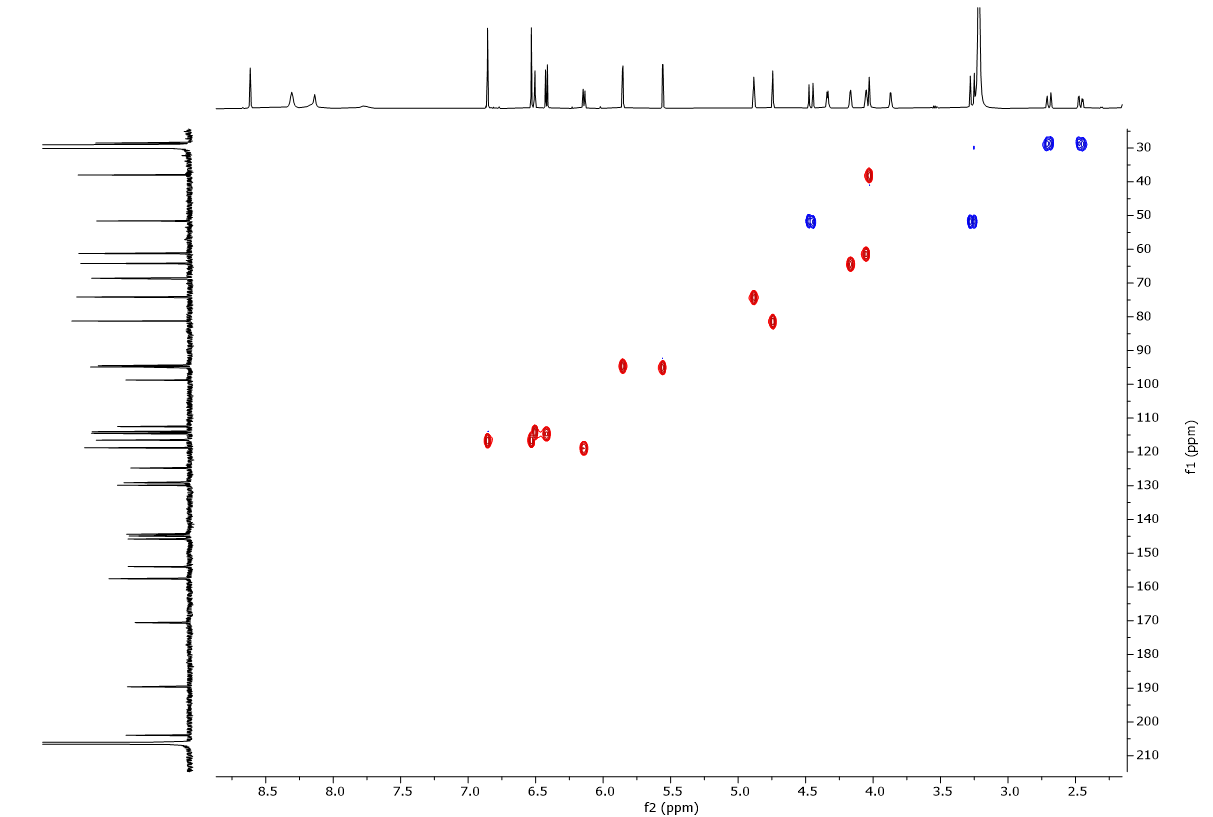


Figure S 20. ^1^H–^13^C HSQC spectrum of the oxidation product 2 of B3 (4) recorded at 14.1 T and 253.0 K in acetone‑*d_6_.*


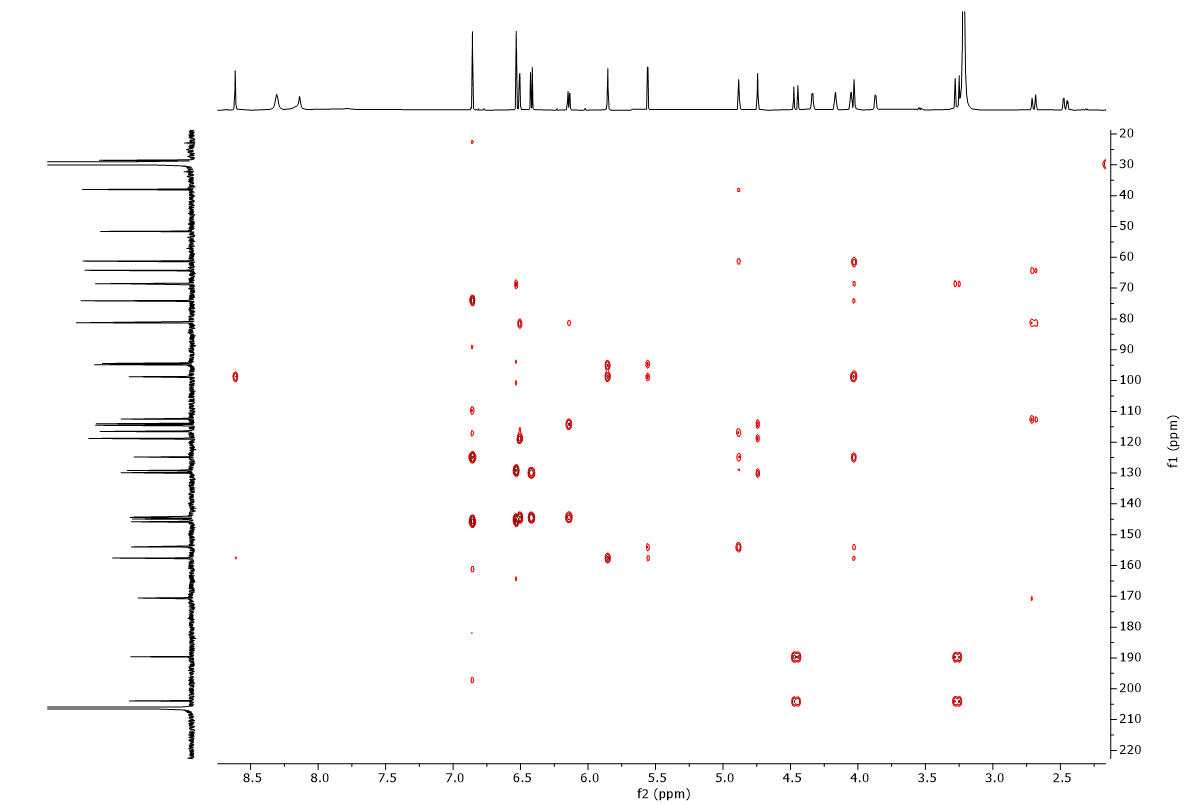


Figure S 21. ^1^H–^13^C HMBC spectrum of the oxidation product 2 of B3 (4) recorded at 14.1 T and 253.0 K in acetone‑*d_6_.*


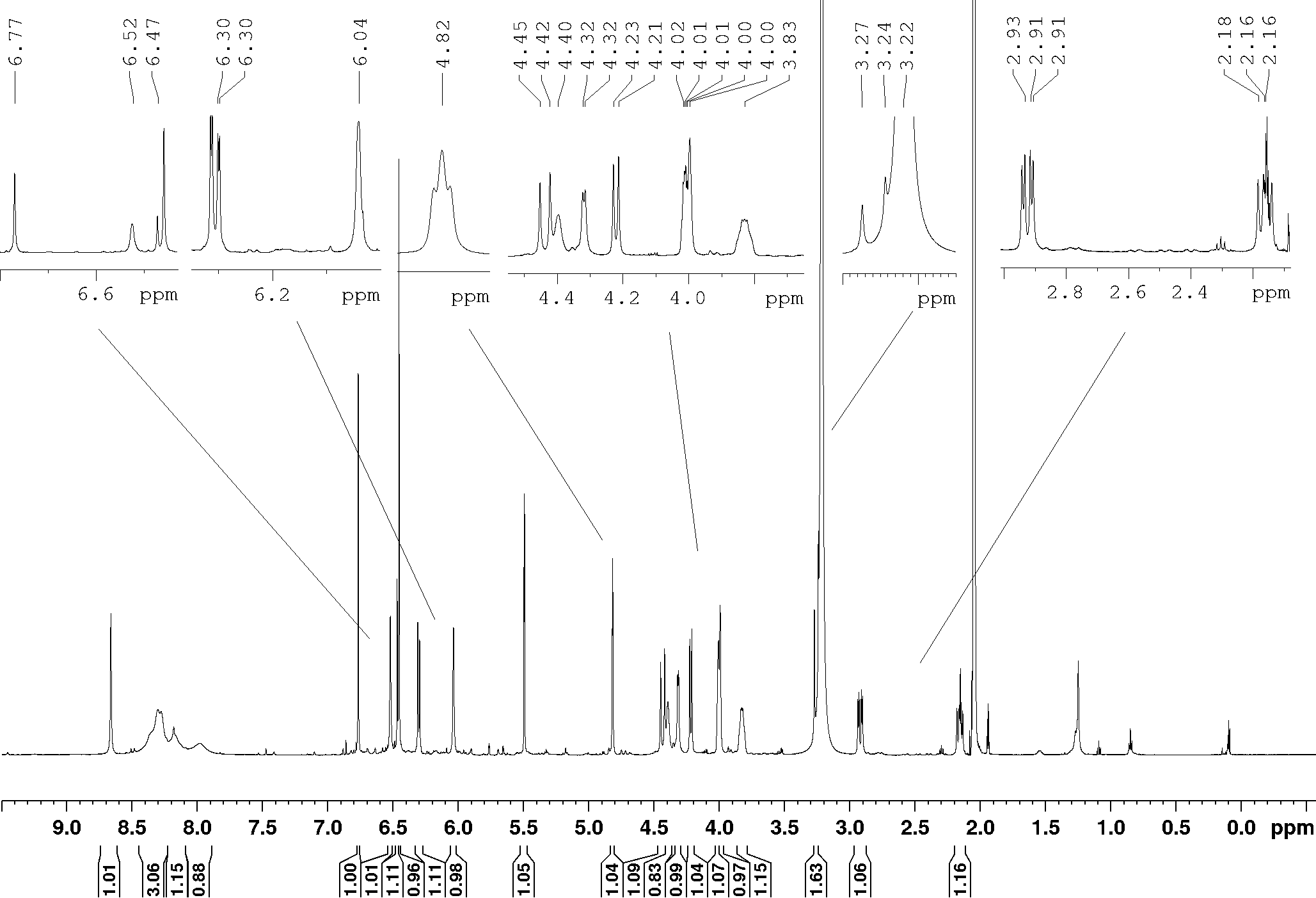


Figure S 22. ^1^H NMR spectrum of the oxidation product of B4 (5) recorded at 14.1 T and 253.0 K in acetone‑*d_6_.*


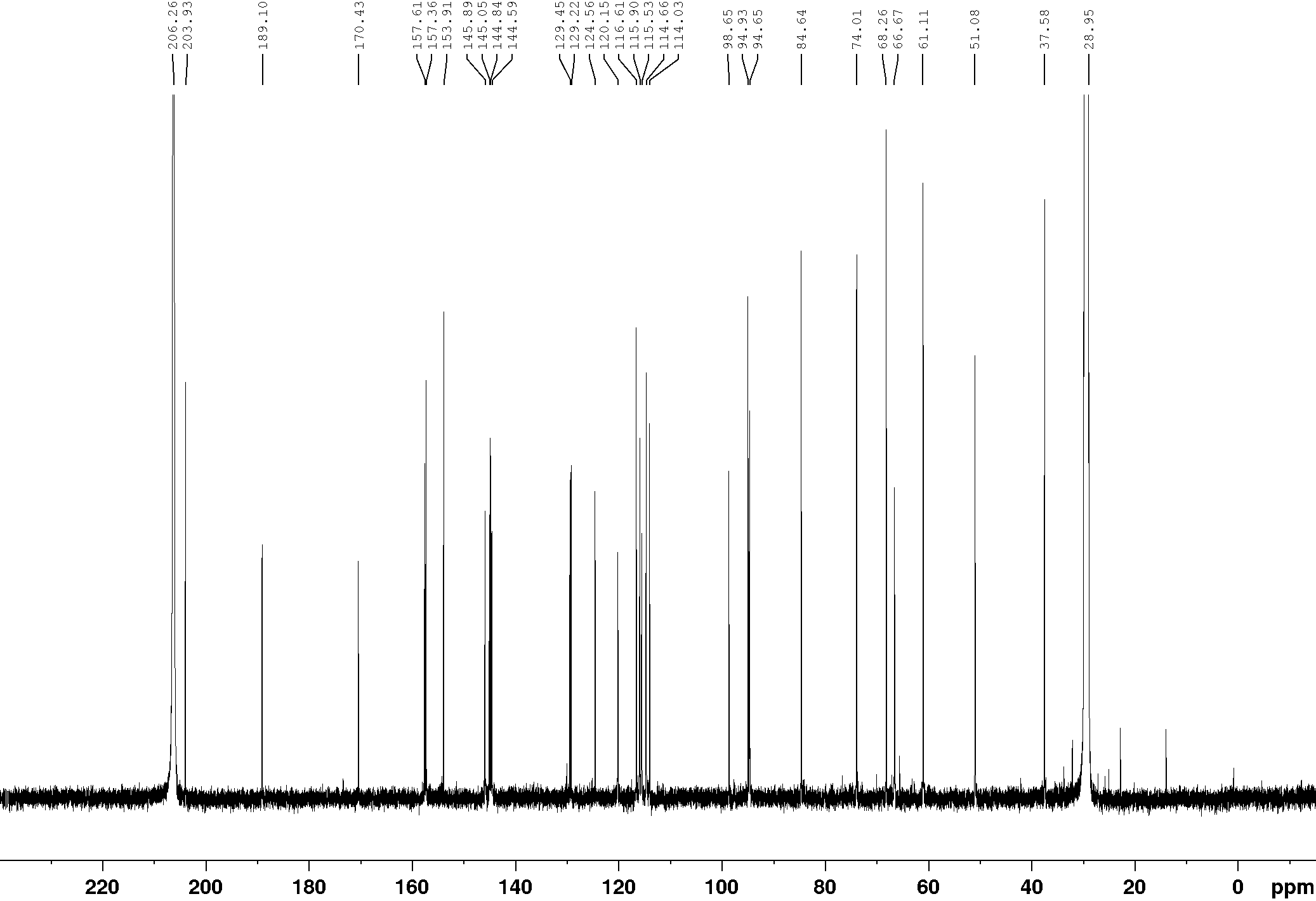


Figure S 23. ^13^C NMR spectrum of the oxidation product of B4 (5) recorded at 14.1 T and 253.0 K in acetone‑*d_6_.*


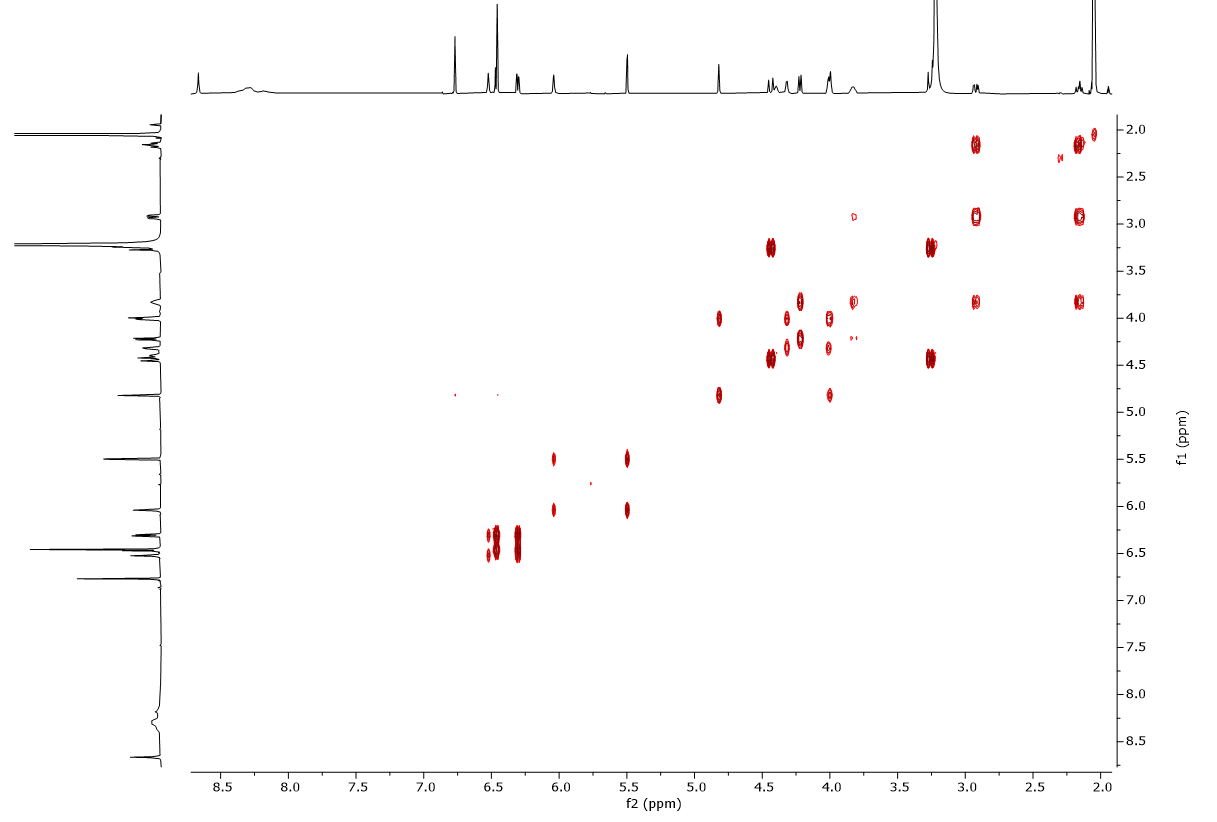


Figure S 24. ^1^H–^1^H COSY spectrum of the oxidation product of B4 (5) recorded at 14.1 T and 253.0 K in acetone‑*d_6_.*


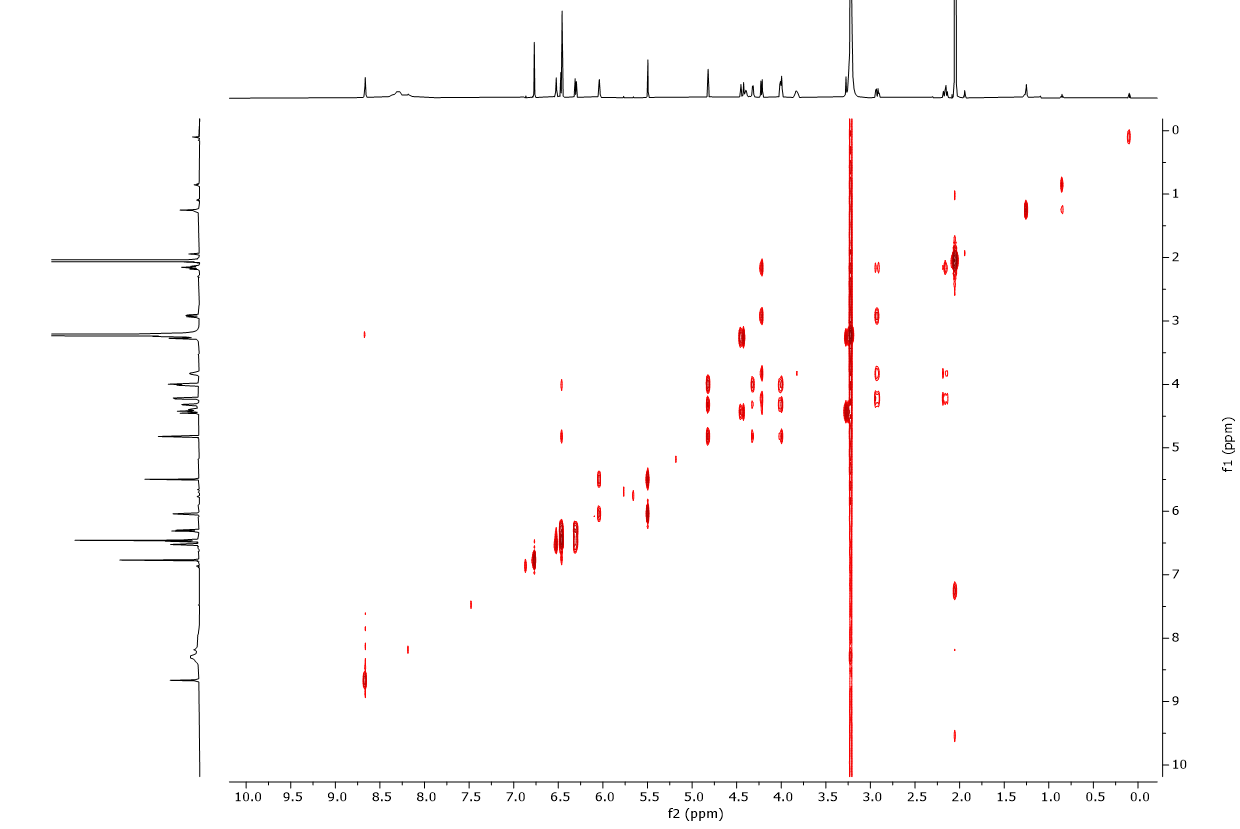


Figure S 25. ^1^H–^1^H TOSY spectrum of the oxidation product of B4 (5) recorded at 14.1 T and 253.0 K in acetone‑*d_6_.*

Figure S 26. ^1^H–^1^H ROESY spectrum of the oxidation product of B4 (5) recorded at 14.1 T and 253.0 K in acetone‑*d_6_.*


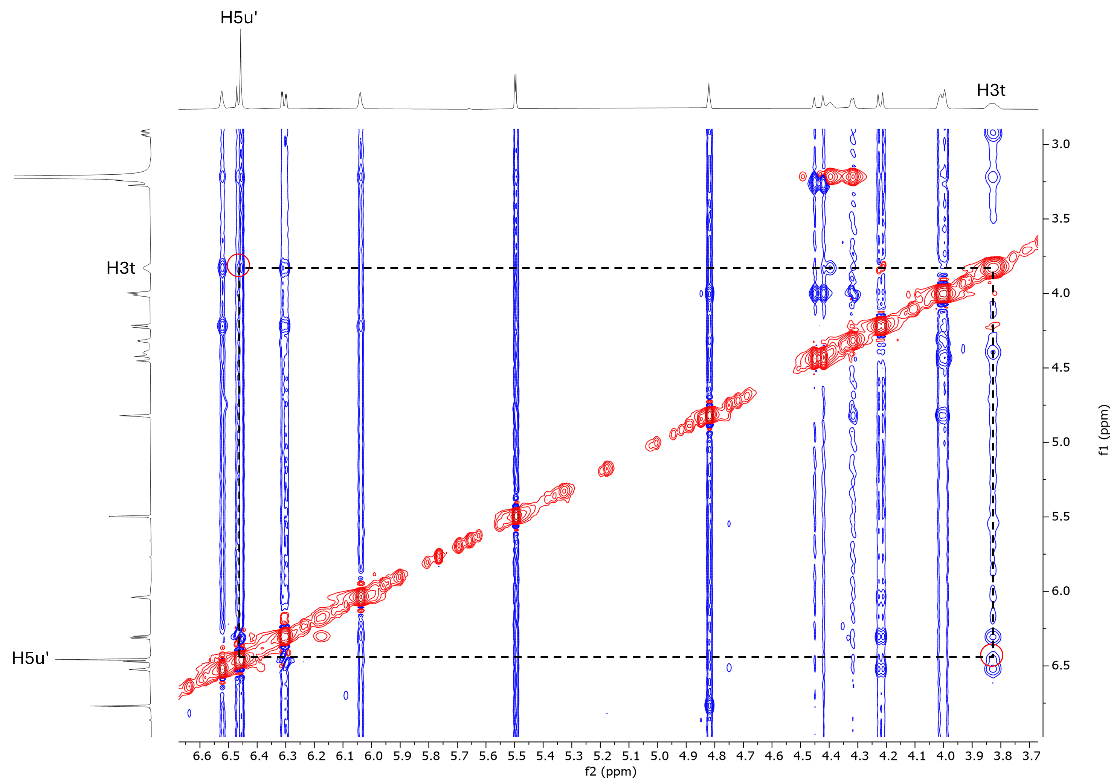


Figure S 27. Partial ^1^H–^1^H ROESY spectrum of the oxidation product 1 of B4 (5) recorded at 14.1 T and 253.0 K in acetone‑*d_6_*, showing the observed ^1^H–^1^H ROESY correlations relevant for the determining of sterochemistry.


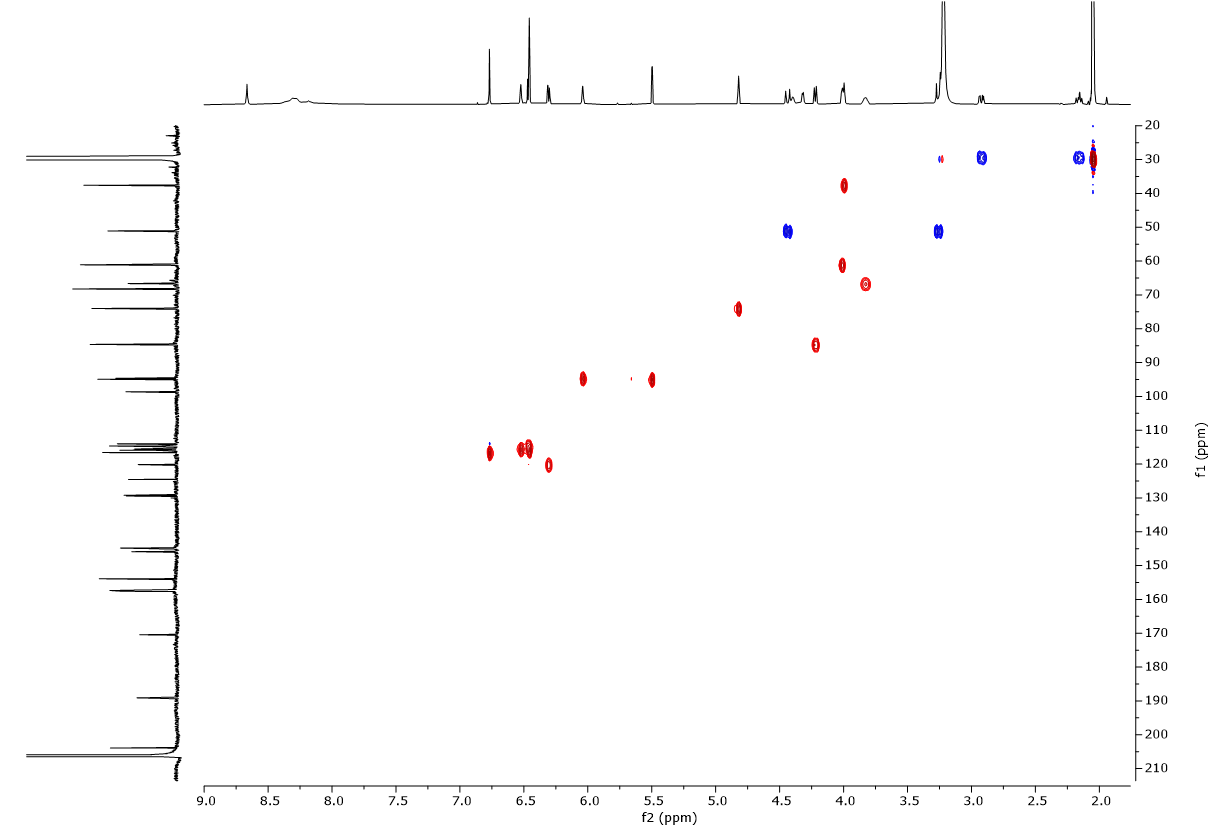


Figure S 28. ^1^H–^13^C HSQC spectrum of the oxidation product of B4 (5) recorded at 14.1 T and 253.0 K in acetone‑*d_6_.*


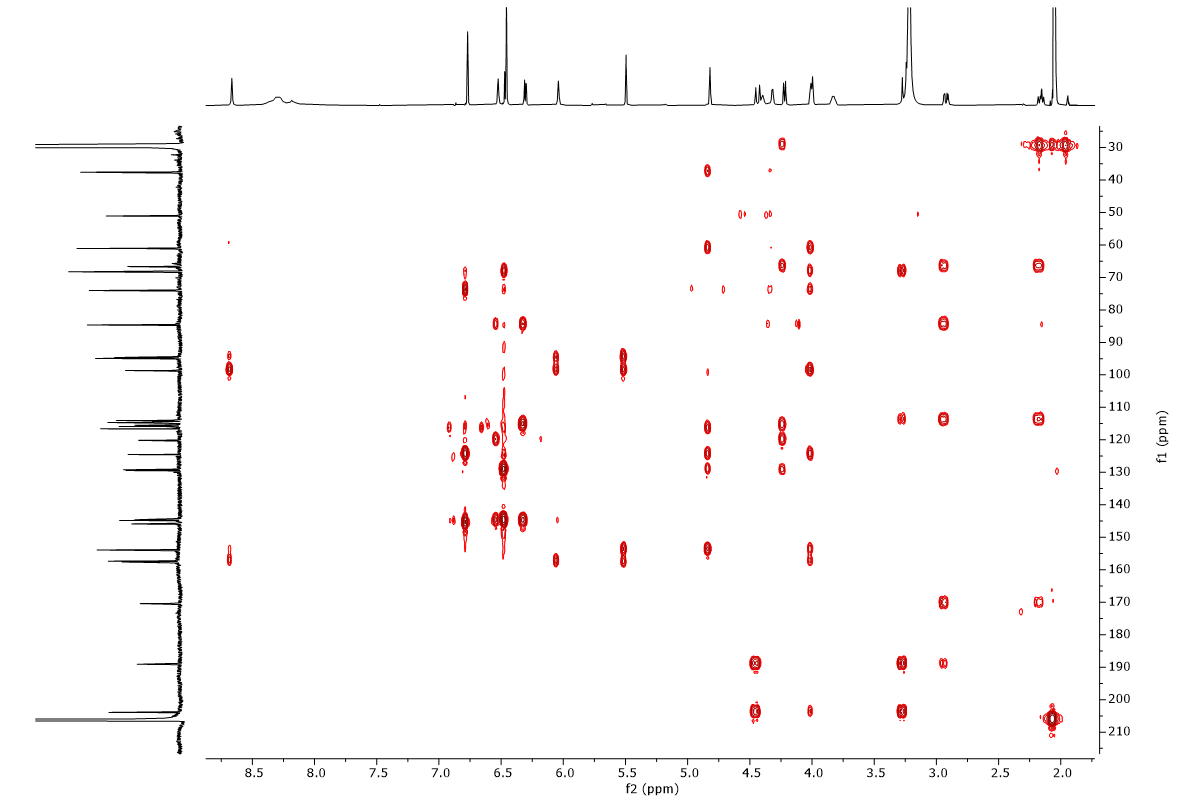


Figure S 29. ^1^H–^13^C HMBC spectrum of the oxidation product 2 of B4 (5) recorded at 14.1 T and 253.0 K in acetone‑*d_6_.*
